# A novel lncRNA ROPM-mediated lipid metabolism governs breast cancer stem cell properties

**DOI:** 10.1186/s13045-021-01194-z

**Published:** 2021-10-29

**Authors:** Shuiqing Liu, Yan Sun, Yixuan Hou, Liping Yang, Xueying Wan, Yilu Qin, Yongcan Liu, Rui Wang, Pengpeng Zhu, Yong Teng, Manran Liu

**Affiliations:** 1 Key Laboratory of Laboratory Medical Diagnostics, Chinese Ministry of Education, Chongqing Medical University, No. 1, Yi-Xue-Yuan Road, Yu-zhong District, Chongqing, 400016 China; 2 Department of Cell Biology and Medical Genetics, Basic Medical School, Chongqing Medical University, Chongqing, 400016 China; 3 Experimental Teaching and Lab Management Center, Chongqing Medical University, Chongqing, 400016 China; 4 Department of Hematology and Medical Oncology, Winship Cancer Institute, Emory University School of Medicine, Atlanta, GA 30322 USA

**Keywords:** Long noncoding RNA, Breast cancer stem cells, PLA2G16, Phospholipid metabolism, Chemo-resistance

## Abstract

**Background:**

Cancer stem cells (CSCs) are considered as the major cause to tumor initiation, recurrence, metastasis, and drug resistance, driving poor clinical outcomes in patients. Long noncoding RNAs (lncRNAs) have emerged as crucial regulators in cancer development and progression. However, limited lncRNAs involved in CSCs have been reported.

**Methods:**

The novel lncROPM (a regulator of phospholipid metabolism) in breast CSCs (BCSCs) was identified by microarray and validated by qRT-PCR in BCSCs from breast cancer cells and tissues. The clinical significance of lncROPM was evaluated in two breast cancer cohorts and TANRIC database (TCGA-BRCA, RNAseq data). Gain- and loss-of-function assays were performed to examine the role of lncROPM on BCSCs both in vitro and in vivo. The regulatory mechanism of lncROPM was investigated by bioinformatics, RNA FISH, RNA pull-down, luciferase reporter assay, and actinomycin D treatment. PLA2G16-mediated phospholipid metabolism was determined by UHPLC-QTOFMS system. Cells’ chemosensitivity was assessed by CCK8 assay.

**Results:**

LncROPM is highly expressed in BCSCs. The enhanced lncROPM exists in clinic breast tumors and other solid tumors and positively correlates with malignant grade/stage and poor prognosis in breast cancer patients. Gain- and loss-of-function studies show that lncROPM is required for the maintenance of BCSCs properties both in vitro and in vivo. Mechanistically, lncROPM regulates PLA2G16 expression by directly binding to 3'-UTR of PLA2G16 to increase the mRNA stability. The increased PLA2G16 significantly promotes phospholipid metabolism and the production of free fatty acid, especially arachidonic acid in BCSCs, thereby activating PI3K/AKT, Wnt/β-catenin, and Hippo/YAP signaling, thus eventually involving in the maintenance of BCSCs stemness. Importantly, lncROPM and PLA2G16 notably contribute to BCSCs chemo-resistance. Administration of BCSCs using clinic therapeutic drugs such as doxorubicin, cisplatin, or tamoxifen combined with Giripladib (an inhibitor of cytoplasmic phospholipase A2) can efficiently eliminate BCSCs and tumorigenesis.

**Conclusions:**

Our study highlights that lncROPM and its target PLA2G16 play crucial roles in sustaining BCSC properties and may serve as a biomarker for BCSCs or other cancer stem cells. Targeting lncROPM-PLA2G16 signaling axis may be a novel therapeutic strategy for patients with breast cancer.

**Supplementary Information:**

The online version contains supplementary material available at 10.1186/s13045-021-01194-z.

## Introduction

Breast cancer is the most common malignancy in women worldwide with the highest incidence [1]. Although considerable advancement has been made in prevention, early diagnose, and individual treatment, breast cancer is still the second major cause of cancer-related death due to recurrence, distant metastasis, and chemo-resistance [2]. Cancer stem cells (CSCs) (also called tumor-initiating cells) are defined as a subpopulation of tumor cells that possess the capacity for self-renewal, tumor initiation, and tumor maintenance [3]. CSCs have been documented in leukemia and many solid tumors, including breast cancer. Moreover, CSCs are considered to be the “seeds” of tumor initiation, development, recurrence, metastasis, and chemo-resistance [4, 5]. Therefore, investigating the mechanism of sustaining CSC characteristics may provide new strategies for breast cancer therapy.

Metabolic reprogramming has become a hallmark of tumor cells since Otto Warburg first observed the Warburg effect in the 1920s [6, 7]. Tumor cells alter metabolic pattern to satisfy energy requirements for maintaining malignant properties [8, 9]. As for CSCs, previous reports have identified that metabolic reprogramming is essential for stem cells fate and behaviors [10, 11]. For example, pancreatic non-CSCs depend on highly glycolytic metabolism, whereas pancreatic CSCs strongly rely on mitochondrial OXPHOS, but when mitochondrial inhibitor metformin was used to treat CSCs, metformin-resistant CSCs reversed the metabolic phenotype toward non-CSCs by increasing MYC expression to enhance glycolytic capacity and promote CSCs survival [12]. It raises that metabolic-related genes play an important role in modulating metabolic reprogramming and provide a novel therapeutic target to eliminate CSCs [13]. However, the complex mechanism underlying the metabolism of CSCs and stemness maintenance are still incompletely understood.

Long noncoding RNAs (lncRNAs) are a large class of transcripts longer than 200 nucleotides with limited protein coding potential [14]. Recently, lncRNAs have become a hotspot owing to their emerging functions in diverse biological processes [15, 16], such as proliferation, apoptosis, metastasis, metabolism, drug resistance, and stemness maintenance. In addition, lncRNAs are one of critical regulators in gene expression by modulating nuclear architecture and transcription in the nucleus and affecting mRNA stability, translation, and posttranslational modifications in the cytoplasm [17]. Although growing evidence shows that lncRNAs are frequently dysregulated in CSCs, only a small number of lncRNAs have been identified functionally. Fortunately, in our previous works, we identified a set of BCSC-specific lncRNAs between breast CSCs and non-CSCs using microarray analysis. A novel metabolic-related lncRNA, termed as lncROPM (a regulator of phospholipid metabolism), which was predicted to target a phospholipid metabolism-associated PLA2G16, aroused our great attention.

PLA2G16 (Group XVI phospholipase A2), also known as PLAAT3, H-REV-107, HRASLS3 (Ha-RAS-like suppressor 3), and AdPLA2 (adipose-specific PLA2), was first considered as a tumor suppressor by inhibiting Ras-mediated transformation in H-RAS-resistant murine fibroblasts [18]. Additionally, PLA2G16 overexpression impeded cells’ proliferation and conduced to apoptosis in ovarian cancer [19, 20]. On the contrary, Irina Nazarenko and his colleagues found that PLA2G16 is not only inducing cells proliferation, driving poor prognosis of patients in non-small cell lung cancer, but also up-regulation in stomach, colon, and rectum cancers, illustrating that PLA2G16 serves as an oncogenic role in these tumors [21]. Interestingly, PLA2G16 was later classified as a member of the phospholipase A2 family and involved in phospholipid metabolism by catalyzing hydrolysis from phospholipids sn-2 ester bond [22]. The sn-2 position of phospholipids is generally rich in arachidonic acid (AA) or other unsaturated fatty acids, which are the important cellular metabolites and can mediate various signal transductions in tumor initiation and progression [23–25]. What’s more, PLA2G16 is overexpressed in adipose tissue and facilitates adipocyte lipolysis, suggesting that PLA2G16 has a great effect on lipid metabolism [26]. To date, increasing data demonstrate that PLA2G16 contributes to tumor growth, metastasis, and drug resistance [27–30]. Nevertheless, whether PLA2G16 contributes to tumor progression through altering lipid metabolism pathways has not been reported in the literature, as well as the function of PLA2G16 in CSCs.

In this study, we first revealed that lncROPM is highly expressed in BCSCs and locates in cytoplasm of BCSCs. Mechanistically, we found that lncROPM up-regulates the expression of PLA2G16 by stabilizing PLA2G16 mRNA to alter phospholipid metabolism and result to abundant production of free fatty acid especially arachidonic acid, which activates PI3K/AKT, Wnt/β-catenin, and Hippo/YAP signaling pathways to contribute CSCs’ features. In addition, lncROPM and PLA2G16 were closely associated with tumor malignancy, recurrence, chemo-resistance, and poor prognosis in clinic breast cancer patients. Interestingly, we discovered that Giripladib, a cytoplasmic phospholipase A2 inhibitor that had already been tested in clinical trials to patients with chronic osteoarthritis [31], can effectively inhibit BCSC characteristics and chemo-resistance. Furthermore, a combination of clinic therapeutic drugs such as doxorubicin, cisplatin, or tamoxifen with Giripladib obviously benefit eliminating CSCs in breast cancer both in vitro and in vivo. Taken all, our work not only sheds light on a pivotal role of lncROPM in regulating CSCs stemness but also provides a novel strategy for the treatment of breast cancer.

## Materials and methods

### Cell culture and reagents

The human breast cancer cells (BT-549, Hs578T, MDA-MB-231, MDA-MB-468, MDA-MB-436, MCF-7, and T47D) were obtained from the American Type Culture Collection (ATCC, USA). All cells were cultured in the recommended medium (Gibco, USA), supplemented with 10% fetal bovine serum (Gibco, USA) and 1% streptomycin/ penicillin (Beyotime, Shanghai, China) at 37 °C with 5% CO_2_. To establish cisplatin (DDP)-resistant MDA-MB-231 cell line (MDA-MB-231/DDP), doxorubicin-resistant BT549 cell line (BT-549/DOX) and tamoxifen-resistant MCF-7 cell line (MCF-7/TAM), MDA-MB-231, BT-549 and MCF-7 cells were exposed to repetitive and incremental concentrations of drugs over a period of 6 months. To maintain the resistance phenotype, MDA-MB-231/DDP, BT-549/DOX, and MCF-7R cells were, respectively, cultured in the presence of 2 μM cisplatin (Med-ChemExpress, NJ, USA), 2 μM doxorubicin (Med-ChemExpress, NJ, USA), and 1 μM tamoxifen (Med-ChemExpress, NJ, USA). Arachidonic acid was obtained from Sigma-Aldrich (St. Louis, MO, USA). Giripladib, an inhibitor of cytoplasm phospholipase A2, was purchased from USBiological Life Sciences (Swampscott, MA, USA).

### Clinical tissue specimens

Human tumor tissues and their corresponding normal tissues (at least 5 cm away from a tumor) were collected from breast cancer patients who received initial surgery and did not accept any preoperative radiotherapy or chemotherapy at the First Affiliated Hospital of Chongqing Medical University. The primary human breast cancer cells were isolated from fresh tumor tissues after resection. All breast tumor samples were numbered at the collecting time point. The primary tumor cells isolated from clinical tumor sample #5 (breast cancer patient with an advanced breast tumor tissue, 57 years old, female, tumor size 4.0 × 3.0 × 2.5 cm, metastasis) and its derived mammospheres were used for subsequent experiments. In addition, chemosensitive and chemoresistant breast tumor tissues were obtained from patients who underwent resection after neoadjuvant chemotherapy. Tumor’s response to neoadjuvant chemotherapy was evaluated according to the Response Evaluation Criteria in Solid Tumors (RECIST) guidelines. The clinical and pathological characteristics of breast cancer patients were retrieved from medical records under institutionally approved protocols. Specimens were fixed in 10% formalin, embedded in paraffin, and cut into 4-μm sections on unstained slides for subsequence analysis. This study was approved by the Ethics Committee of Chongqing Medical University.

### MACS for breast cancer stem cells (BCSCs)

CD44/CD24 antibodies conjugated to magnetic microbeads (Miltenyi Biotec, Bergisch Gladbach, Germany) were used to obtain the BCSCs and non-BCSCs from breast cancer cells. The isolation process followed the manufacturer's instructions.

### RNA preparation and quantitative reverse-transcription PCR (qRT-PCR)

Total RNA was isolated from tissue specimens and cancer cells by TRIzol reagent (Takara, Japan) according to the manufacturer's instructions and then performed to reverse transcription with the PrimeScript RT Reagent Kit (Takara, Japan). Gene expression was detected by qRT-PCR using the SYBR Premix Ex Taq II kit (Takara, Japan). The relative expression of RNAs was calculated using the comparative Ct method and normalized to β-actin transcripts. All the experiments were conducted at least three times. The primers used are listed in Additional file 2: Table 1.

### Mammosphere formation and self-renewal capability assay

Mammosphere culture was performed as previously described [32]. Breast CSC-like cells (CD44^+^CD24^−/low^) and non-CSC (CD44^−/low^CD24^+^) cells isolated from BT-549, Hs578T, MDA-MB-231, MDA-MB-468, MDA-MB-436, MCF-7, T47D, and tumor sample cells were plated in six-well plates coated with 2% poly-2-hydroxyethyl methacrylate (poly-HEMA, Sigma-Aldrich, USA) at a density of 1×10^4^ cells/ml and 5 × 10^3^ cells/ml in following passages. Cells were cultured in a serum-free DMEM/F12 (Gibco, USA) medium supplemented with B27 (Gibco, USA), 20 ng/ml epidermal growth factor (EGF, Invitrogen), 20 ng/ml basic fibroblast growth factor (bFGF, Invitrogen, USA), 0.4% albumin from bovine serum (BSA, Sigma-Aldrich, USA), 2 μg/ml heparin (Sigma-Aldrich, USA), and insulin-transferrin-selenium (Invitrogen, USA). Mammospheres were incubated at 37 °C with 5% CO_2_ and passaged every 7 days. To test the self-renewal capability, the number and size of mammospheres (diameter > 50 µm) were counted manually, and representative images were obtained using an OLYMPUS IX70 microscope (Tokyo, Japan). The percentage of mammosphere-forming efficiency (MFE) was calculated using the following equation: (number of mammospheres per well/number of cells seeded per well) × 100. The average size of mammospheres (*N* = 30 spheres) was calculated.

### RNA fluorescence in situ hybridization (FISH)

For in situ detection of lncROPM in breast cancer cells or tumor tissues, Cy3-labeled lncROPM probes, U6 probes, and 18S probes were designed and synthesized by RiboBio (China). Cell or tissue FISH assay was performed with a Fluorescence in situ Hybridization Kit (RiboBio, China) according to the manufacturer's instructions. Confocal laser scanning microscopy (Leica, Germany) was used to observe the images.

## Subcellular fractionation

To determine the cellular localization of lncROPM, cytoplasmic and nuclear RNA were isolated using PAKIS Kit (Thermo Fisher Scientific, USA) according to the manufacturer's instructions. Extracted RNAs were reverse-transcribed immediately, and the expression of genes was measured by qRT-PCR analysis. The U6 RNA was used as nuclear control and GAPDH mRNA as cytoplasmic control.

### Establishment of stable expression cell lines

In order to knock down the expression of lncROPM and PLA2G16, the specific shRNA against the target gene and control shRNA synthesized by GenePharma (Shanghai, China) was separately inserted into the pGLV3/H1/GFP/Puro vector from GenePharma (Shanghai, China). The detail shRNA sequences are listed in Additional file 2: Table 1. For overexpression analysis, lncROPM and PLA2G16 sequences synthesized by GenePharma (Shanghai, China) were cloned into lentiviral expression vector LV5/EF-1aF/GFP/Puro from GenePharma (Shanghai, China). The packaged lentivirus was used to infect breast cancer cells following the manufacturer's instructions. To establish the stably engineered cells, the infected cells were treated with 1 µg/ml puromycin (Gibco, USA) for two weeks.

### Western blotting analysis

Briefly, breast cancer cells were harvested, washed, and lysed in RIPA buffer (Beyotime, China) containing protease inhibitor cocktail (Beyotime, China). Total protein was extracted and quantified with BCA protein assay kit (Beyotime, China). Equal amounts of proteins were separated by 8%–12% SDS-PAGE gel and transferred to PVDF membranes (Bio-Rad, USA). The membranes were blocked in 5% non-fat milk (BOSTER, China) for 2 h and incubated with primary antibody overnight at 4 °C. After washing with TBST, the membranes were subsequently incubated with the appropriate horseradish peroxidase (HRP)-conjugated anti-mouse or rabbit IgG (ZSGB-BIO, China) for 1 h at room temperature. The protein bands were visualized and detected by the enhanced chemiluminescence system (Bio-Rad, Hercules, EDA USA). Images were captured using Scion image software. β-Actin was utilized as an internal control. The following primary antibodies were used in this study: KLF4 (1:500, 1880-1-AP, Proteintech), SOX2 (1:500, ab97959, Abcam), OCT4 (1:2000, 60242-1-Ig, Proteintech), PLA2G16 (1:500, A16018, ABclonal), Akt (1:500, 10176-2-AP, Proteintech), p-Akt (Ser473) (1:500, 66444-1-Ig, Proteintech), PI3K (1:500, CY5355, Abways), p-PI3K (Tyr607) (1:500, CY6427, Abways), YAP1 (1:500, CY5381, Abways), p-YAP1 (S127) (1:500, CY5743, Abways), β-Catenin (1:500, A5038, Bimake), Notch1 (1:500, A5176, Bimake), Shh (1:500, A5115, Bimake), PARP (1:500, A5037, Bimake), GAPDH (1:500, 40493-1, SAB), β-Actin (1:1000, BA2305, BOSTER).

### Flow cytometry analysis

To detect the BCSC subpopulations, the following antibodies were used: APC-anti-CD44 (eBioscience, 17–0441, 1:167 dilution), FITC-anti-CD24 (eBioscience, 11–0247, 1:20 dilution), APC-rat isotype control (eBioscience, 174,031, 1:167 dilution), FITC-mouse isotype control (eBioscience, 11–4714, 1:20 dilution). A total of 1 × 10^6^ cells were incubated with antibodies in the dark at 4 °C for 30 min. Cells were washed and re-suspended in 500 µl of PBS and analyzed using a flow cytometer (Beckman Coulter, High Wycombe, UK).

### In vitro limiting dilution assay

Cells were plated into 96-well ultra-low attachment culture dishes at dose of 1000, 100, and 10 cells per well in 24 replicates and incubated in mammosphere-forming conditions for 7 days. Wells containing mammosphere were counted. Data were calculated and compared using the Extreme Limiting Dilution Analysis (http://bioinf.wehi.edu.au/ software/elda/).

### In vivo limiting dilution assay

Animal procedures were approved by the animal care ethics committees at Chongqing Medical University. Various amounts (1 × 10^3^, 10^4^, 10^5^) of cells were collected in 100 µl of PBS:Matrigel at a 1:1 ratio and subcutaneously injected into one inguinal mammary fat pad of 5-week-old female nude mice (five mice per group). Next, the mice were monitored every 5 days for 2 months and then killed by cervical dislocation. Tumor-initiating potential was assessed as the ability to form a palpable tumor mass > 0.1 mm^3^ volume. Frequency of stem cells was determined using the Extreme Limiting Dilution Analysis (http://bioinf.wehi.edu. au/software/elda/).

### Cell viability assay

Cell viability was detected by using the Cell Counting Kit-8 (CCK8, Dojindo, Japan) following the manufacturer's protocol. In brief, cells were seeded into 96-well plates at a density of 2000 cells per well overnight at 37 °C. Subsequently, cells were treated with different concentrations of cisplatin, doxorubicin or tamoxifen for 48 h, and then, 10 µl of CCK-8 solution was added to each cell of the plate. After 3 h of incubation, the plates were read at 450 nm by a microplate reader (BioTek, Winooski, Vermont, USA). All experiments were conducted in triplicate.

### LncRNA target prediction

Cis- and trans-analyses were used to predict the potential target of lncRNA. To identify lncRNA cis-regulated target genes, lncRNA and its potential target genes were paired and visualized using UCSC genome browser (http://genome.ucsc.edu/). A gene closest to lncRNA within a 10-kb region upstream or downstream was regarded as a target gene of the lncRNA [33]. To identify lncRNA trans-regulated target genes, blast alignment was carried out for the first round screening based on the principle of sequence complementary pairing. Then, RNAplex software was employed to choose trans-acting target genes [34].

### RNA stability analysis

Cells were exposed to 2 µg/ml transcription inhibitor actinomycin D (Cayman Chemical, USA) for 0, 2, 4, 6, or 8 h to block transcription, and DMSO (Sigma-Aldrich, USA) was used as the control reagent. Cell samples were collected at the indicated time points followed by RNA extraction, and then, qRT-PCR was performed to analyze PLA2G16 mRNA expression levels. RNA stability was described as the percentage of remaining RNA at the recommended time after addition of Act-D relative to the RNA content at the 0 h time point.

### RNA pull-down

RNA pull-down assays were performed with the Pierce™ Magnetic RNA–Protein Pull-Down kit (Thermo Fisher Scientific) according to the manufacturer's instructions. Briefly, desthiobiotinylated RNA was incubated with cell lysates associated with streptavidin-linked magnetic beads. The binding RNA was detected by qRT-PCR analysis. The biotin-labeled RNA probes are listed in Additional file 2: Table 1. All processes were conducted under RNase-free conditions.

### Luciferase reporter assay

The sequences of PLA2G16 3'-UTR-1 and 3'-UTR-2 were inserted into PMIR-Reporter vector to obtain PMIR-PLA2G16-3'-UTR-1 and PMIR-PLA2G16-3'-UTR-2 reporter. Then, PMIR-PLA2G16-3'-UTR-1 or PMIR-PLA2G16-3'-UTR-2 was transfected into lncROPM-knockdown BCSCs and lncROPM-overexpressing non-BCSCs, respectively, by using lipofectamine 2000 (Invitrogen, USA). Similarly, PMIR-PLA2G16-3'-UTR-1 and overexpression plasmid with lncROPM-truncated fragment were co-transfected into 293 T cells. As an internal transfection control, pRL-TK Renilla luciferase reporter vector was also co-transfected into each sample. After 30 h of incubation, luciferase activity was measured with a Dual-Luciferase Reporter System (Promega, USA).

### Lipid extraction

Lipids were extracted from cells according to the method described by Rahul Vijay Kapoore et al. [35]. Briefly, cells (1 × 10^7^/experimental group) were washed twice with phosphate-buffered saline (PBS, pH 7.4) and rapidly quenched by the addition of fivefold volumes of precooled 60% aqueous methanol supplemented with 0.85% (w/v) ammonium bicarbonate. The quenched biomass was centrifuged at 2500 × g for 5 min at − 9 °C. Subsequently, the supernatant was removed. Then, cells pellets were collected, rapidly snap frozen in liquid nitrogen, and stored at − 80 °C for further analysis.

### Lipid analysis by UHPLC-QTOFMS

Extracted lipids were detected by Ultra High Performance Liquid Tandem Chromatography Quadrupole Time of Flight Mass Spectrometry System (UHPLC-QTOFMS, Agilent 1290 UHPLC + AB Triple TOF 6600 +), and C18 chromatographic column was purchased from Kinetex (Phenomenex, USA; 2.1 × 100 mm, 1.7 µm). Mobile phase conditions were: A: 10 mM HCOONH4 in ACN/H2O (v/v, 6:4), B: 10 mM HCOONH4 in ACN/IPA (v/v, 1:9); Program settings were: 0–12.0 min, 300 µl/min, 40%-100% B; 12.0–13.5 min, 300 µl/min, 100% B; 13.5 ~ 13.7 min, 300 µl/min, 100%-40% B; 13.7–18.0 min, 300 µl/min, 40% B; MS parameters were: Gas1: 60 psi; Gas2: 60 psi; Curtain Gas: 30 psi; Temperature: 600 °C; Ion Spray Voltage Floating: 5000 V; TOF Masses (Da): Min = 200.0000, Max = 1200.0000. Lipids were identified by exact mass and MS/MS spectral match based on in-house database.

### Immunohistochemical staining

Paraffin-embedded sections were de-waxed, rehydrated, and blocked for endogenous peroxidase and nonspecific binding sites. Then, the sections were incubated with rabbit anti-PLA2G16 (1:100, Sangon Biotech), KLF4 (1:200, Proteintech) polyclonal antibody overnight at 4 °C. Subsequently, the slides were incubated with polyperoxidase-anti-rabbit IgG (ZSGB-BIO, China) for 30 min at 37 °C and then stained with diaminobenzidine. After counterstained by hematoxylin and sealed, images were captured using a Nikon Eclipse 80i microscope (Tokyo, Japan).

### Xenograft formation of mammosphere cells

Animal experiments were authorized by the animal care ethics committees of Chongqing Medical University. Mammosphere cells (1 × 10^5^) from BT-549/shCtrl and BT-549/shlncROPM cells were subcutaneously inoculated in the mammary fat pad of 5-week-old female nude mice (five mice per group). When the tumor volume was around 100 mm^3^, the mice were separately intraperitoneally administered with 4 mg/kg doxorubicin every 5 days (1 group), or 7.5 mg/kg Giripladib once daily (1 group), or combination of doxorubicin with Giripladib (1 group). Control mice were treated with sterile saline (1 group). Xenografts were measured every 5 days using a digital caliper, and tumor volumes were calculated using the following formula: volume = (length × width^2^)/2. Three weeks after injection, mice were euthanized and tumors were isolated, weighed, and photographed. Subsequently, tumor tissues were fixed in 10% formalin, embedded in paraffin, and cut in 4-µm sections for immunohistochemistry analysis.

### Statistical analysis

Statistical analysis was performed with GraphPad Prism 7.0 software (San Diego, CA, USA). All experiments were repeated at least three times in parallel, and data were presented as mean ± SD. Two-tailed Student's t test was conducted for statistical significance analysis between two groups, and one-way analysis of variance (ANOVA) was performed for multiple groups. Mann–Whitney test was used to estimate the expression levels of lncROPM and PLA2G16 in different groups of clinical specimens. Pearson correlation analysis was employed to identify the correlation between variables. *p* < 0.05 was assumed as statistically significant.

## Results

### The novel metabolism-related lncROPM is highly expressed in BCSCs

**Fig. 1:**
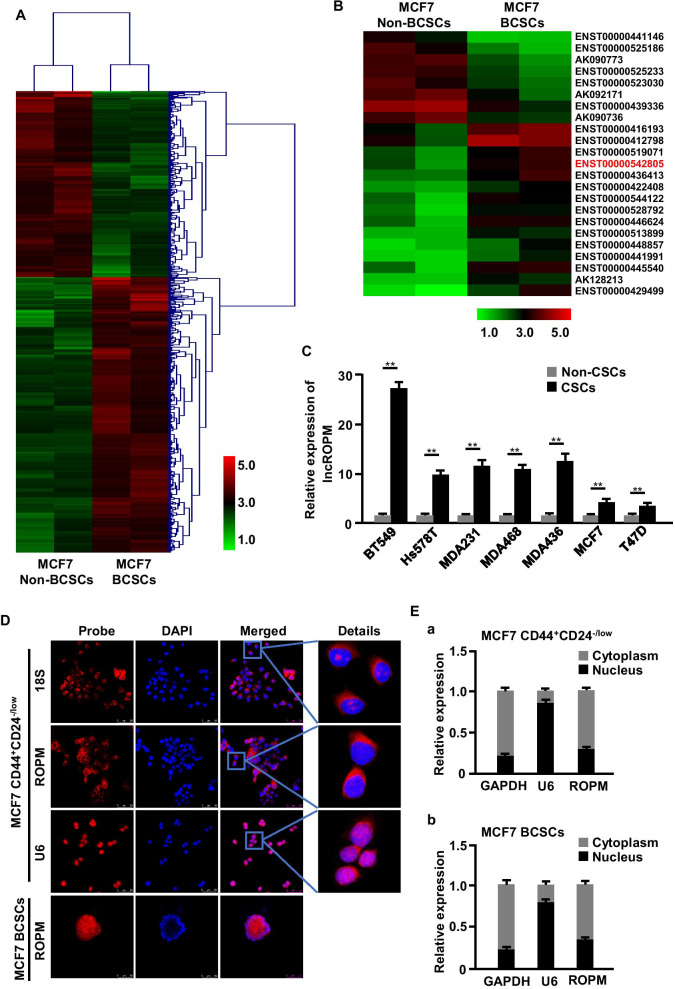
The novel metabolism-related lncROPM is highly expressed in BCSCs. **A** Cluster heat maps of differentially expressed lncRNAs (fold-change > 2, *p*-value < 0.05) between non-BCSCs (CD44^−/low^CD24^+^ cells) and BCSCs (CD44^+^CD24^−/low^ mammospheres) from MCF7 cells. **B** Heat maps of metabolic pathway-related lncRNAs. **C** qRT-PCR analysis of lncROPM expression in non-CSCs and CSCs from various breast cancer cells. **D** Localization of lncROPM in MCF7 CD44^+^CD24^−/low^ cells and CSCs was detected by RNA-FISH analysis (Original magnification, × 200, Scale bar, 50 μm). 18S and U6 served as a positive control for cytoplasmic and nuclear fractions, respectively. The nuclei were stained with DAPI. Fluorescent staining: DAPI (blue), lncROPM (red), 18S (red), U6 (red). **E** Subcellular localization of lncROPM in MCF7 CD44^+^CD24^−/low^ cells and CSCs was measured by qRT-PCR analysis. GAPDH and U6 mRNA were used as a control for cytoplasmic and nuclear transcripts, respectively. The data are presented as the mean ± SD, *p < 0.05, **p < 0.01

It has been proposed that CD44^+^CD24^−/low^ breast cancer cells have the stem/progenitor cell properties, which is called breast CSC-like cells or breast cancer stem cells (BCSCs) [36, 37]. In this study, highly purified BCSCs (CD44^+^CD24^−/low^) and non-BCSCs (CD44^−/low^CD24^+^) were mainly collected through magnetic bead separation (Additional file 1: Figure S1A) and mammosphere enrichment under suspension culture conditions for assessing CSC characteristics. To investigate whether lncRNAs involved in regulating breast cancer stem cell characteristics, we utilized microarray analysis to compare lncRNA expression profiles between non-BCSCs (CD44^−/low^CD24^+^ cells) and BCSCs (CD44^+^CD24^−/low^ mammospheres) from MCF7 cells. As shown in Fig. 1A, a total of 585 lncRNA transcripts were found to be differentially expressed with a cutoff threshold of fold change > 2.0 and *p* < 0.05, among which 349 were upregulated, while 236 downregulated. To confirm the data acquired by microarray, a random subset of upregulated lncRNAs (*n* = 10) and downregulated lncRNAs (*n* = 10) were chosen for qRT-PCR analysis, and results showed that PCR fold changes correlated with the microarray data (Additional file 1: Figure S1B).

Metabolic reprogramming has been reported to play an essential role in stem cells fate and behaviors [38]. Using bioinformatics analysis, we predicted 23 metabolic pathway-related lncRNAs in BCSCs (Fig. 1B). Of them, an uncharacterized lncRNA (Ensembl ID: ENST00000542805), which was highly expressed in BCSCs, located at chromosome 11q12.3-q13.1 in humans with the nearby gene PLA2G16 (Additional file 1: Figure S1C), termed as lncROPM (a regulator of phospholipid metabolism) here, attracted our attention. LncROPM consisted of two exons with a full length of 573 bp, and the noncoding nature was confirmed by coding potential analysis (Additional file 1: Figure S2A-S2B). Next, we examined its level in various breast cancer cells, as well as BCSCs and non-BCSCs from that by qRT-PCR analysis. The results showed that lncROPM was expressed in all the analyzed breast cancer cells. Interestingly, we also found that lncROPM was much higher expressed in the highly invasive cells (MDA-MB-231, Hs578T, BT549, MDA-MB-436) than lowly invasive cell lines (MDA-MB-453, MCF7, T47D, BT474) (Additional file 1: Figure S2C). Subsequently, we separated invaded cells from lowly invasive cell lines (MCF7, T47D, BT474) by Transwell chambers and compared the expression of lncROPM in invaded cells and their parental cells. As shown in Figure S2D-S2E, compared with parental cells, lncROPM was highly expressed in these invaded cells, which also had higher CD44^+^/CD24^−/low^ subpopulation cells than their parental cells. Similarly, lncROPM was much higher in BCSCs than that in non-BCSCs (Fig. 1C). These results suggest that lncROPM may be associated with malignancy of breast cancer and plays a role in BCSCs. Besides, both RNA fluorescence in situ hybridization (FISH) and subcellular fractionation experiments revealed that lncROPM was mainly present in the cytoplasm of BCSCs (Fig. 1D, E and Additional file 1: Figure S2F-S2G).

### LncROPM acts an oncogenic role and closely correlates with clinical characteristics as well as stemness in breast tumors

**Fig. 2:**
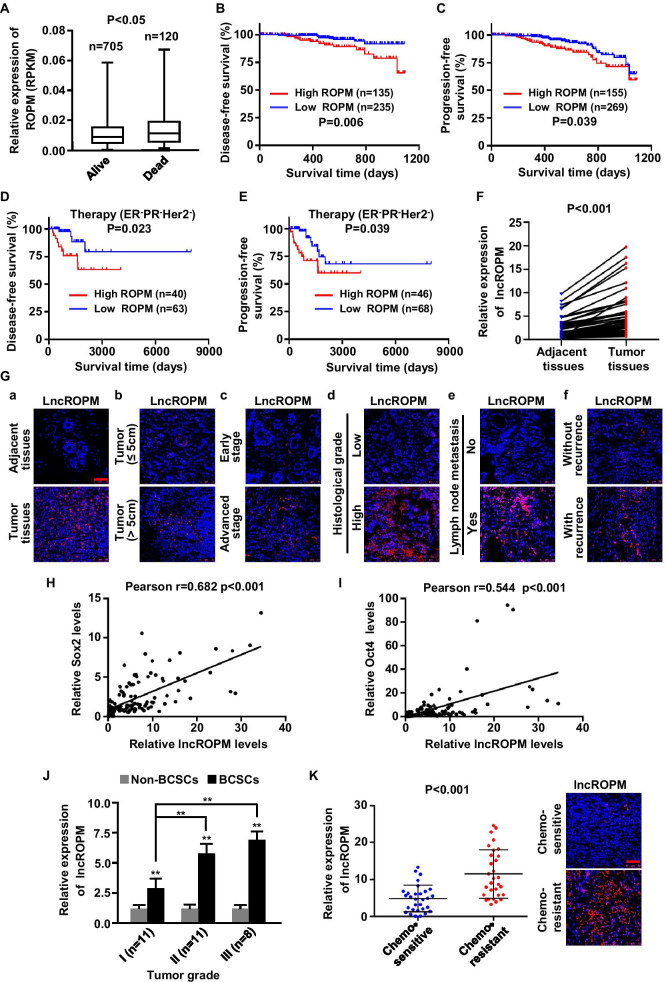
LncROPM is an oncogene and correlated with clinical characteristics as well as stemness in patients with breast cancer. **A** The expression of lncROPM in patients who died of breast cancer (*n* = 120) and surviving from it (*n* = 705) was analyzed using the TCGA-BRCA database (RNAseq data). **B**, **C** Kaplan–Meier survival curve to assess 3-year disease-free survival (**B**) and progression-free survival (**C**) in breast cancer patients with high or low lncROPM expression based on TCGA-BRCA database. **D**, **E** Kaplan–Meier survival analysis of disease-free survival (**D**) and progression-free survival (**E**) according to the lncROPM expression levels in ER^−^PR^−^Her2^−^ breast cancer patients who received clinic treatment based on TCGA-BRCA database. **F** qRT-PCR analysis of lncROPM expression in 50 paired breast cancer tissues and adjacent normal tissues. **G** Representative RNA-FISH staining of lncROPM in breast cancer tissues. The nuclei were stained with DAPI. Fluorescent staining: DAPI (blue), lncROPM (red). **H**, **I** Pearson analysis for the correlation between lncROPM and SOX2 expression levels (**H**) or OCT4 expression levels (**I**) in 130 breast cancer tissues. **J** qRT-PCR analysis of lncROPM expression in non-BCSCs and BCSCs from different breast tumor grade samples. **K** qRT-PCR analysis of lncROPM expression in breast cancer chemosensitive tissues (*n* = 30) and chemoresistant tissues (*n* = 30) (left panel). Representative RNA-FISH staining of lncROPM in tissues was shown in the right panel. The data are presented as the mean ± SD, *p < 0.05, **p < 0.01

To further identify the clinical significance of lncROPM in breast cancer, we first examined its expression in patients from TCGA-BRCA database (RNAseq data). As shown in Fig. 2A, the average level of lncROPM in patients who died of breast cancer (*n* = 120) was higher than that in patients who are surviving (*n* = 705) (*p* < 0.05) (Fig. 2A). Kaplan–Meier survival analysis revealed that high level of lncROPM was significantly related to poor prognosis of patients, based on 3-year disease-free survival and poor progression-free survival (Fig. 2B, C). Moreover, the expression of lncROPM in ER^−^PR^−^Her2^−^ patients who received treatment was negatively correlated with disease-free survival rate and progression-free survival rate (Fig. 2D, E), indicating that lncROPM is related to drug resistance.

Subsequently, we measured lncROPM expression in a cohort of 50 paired breast cancer tissues and adjacent normal tissues by qRT-PCR assays. As shown in Fig. 2F, compared with the adjacent normal tissues, lncROPM was significantly upregulated in breast cancer tissues. In another cohort of 130 breast cancer cases, we assessed the correlation of lncROPM expression with clinical pathology factors. As shown in Table 1, there was a significant association between lncROPM expression and tumor size (*p* = 0.016), TNM stage (*p* = 0.002), histology grade (*p* = 0.019), lymph node metastasis (*p* = 0.042), and tumor recurrence (*p* = 0.001). Representative images are shown in Fig. 2G. It is well known that CSC stemness in tumors is related to the oncogenic dedifferentiation reflected by histological grade. Therefore, we analyzed the relationship between lncROPM expression and stemness markers in breast tumor tissues. Remarkably, lncROPM was positively correlated with Sox2, Oct4, and ALDH1A1 expression in breast tumor tissues (Fig. 2H, I and Additional file 1: Figure S3A). Meanwhile, we assessed lncROPM expression in non-BCSCs and BCSCs from primary breast cancer cells by qRT-PCR, and the results showed that lncROPM was obviously increased in BCSCs compared with non-BCSCs, especially in high tumor grade group (Fig. 2J). The positive correlation expression pattern of lncROPM and Oct4 was also confirmed in BCSCs derived from clinical samples (Additional file 1: Figure S3B). Furthermore, we observed that lncROPM was significantly elevated in chemoresistant breast tumor tissues relative to chemosensitive tumor tissues (Fig. 2K).

**Table 1:** Correlation between lncROPM expression and clinicopathological features in 130 breast cancer patients

Clinicopathological features	Group	No. of patients, *N* = 130 (%)	*p* value
Age (years)	< 50	72 (55.4)	
	≥ 50	58 (44.6)	*p* = 0.575
Tumor size (cm)	< 3	67 (51.5)	
	≥ 3	63 (48.5)	*p* = 0.016*
TNM stage	I–II	70 (53.8)	
	III–IV	60 (46.2)	*p* = 0.002**
Histology grade	I	28 (21.5)	
	II–III	102 (78.5)	*p* = 0.019*
Lymph node metastasis	No	54 (41.5)	
	Yes	76 (58.5)	*p* = 0.042*
Recurrence	No	98 (75.4)	
	Yes	32 (24.6)	*p* = 0.001**

Differences between experimental groups were analyzed by Mann–Whitney test. Data represent mean ± SD (*p < 0.05; **p < 0.01)

Interestingly, by analyzing TCGA database, we also found that lncROPM had important functions in other cancers. For example, in kidney renal clear cell carcinoma (KIRC), high expression of lncROPM was strongly associated with poor survival outcomes (*n* = 515, *p* = 0.003) (Additional file 1: Figure S3C). In head and neck squamous cell carcinoma (HNSCC) and ovarian serous cystadenocarcinoma (OV), lncROPM was apparently increased with the histological grade (Additional file 1: Figure S3D-S3E). Next, we examined lncROPM expression in CSCs from KIRC, HNSCC, and OV by qRT-PCR, and the results displayed that lncROPM was notably high-expressed in CSCs compared with non-CSCs (Additional file 1: Figure S3F-S3H). Collectively, these data indicate that lncROPM acts as an oncogenic lncRNA in breast cancer and other cancers and may play an important role in promoting breast cancer initiation and development.

### LncROPM is required for the maintenance of BCSC properties

**Fig. 3:**
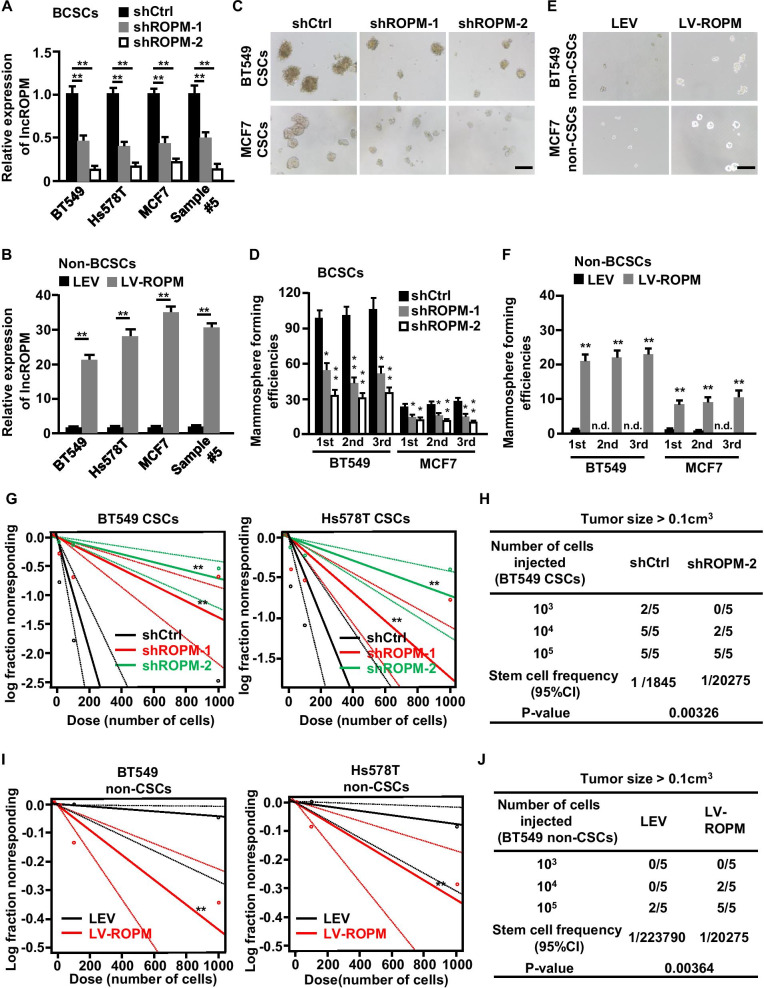
LncROPM is required for the maintenance of BCSCs properties. **A**, **B** qRT-PCR analysis of lncROPM expression in BCSCs transfected with shCtrl (shControl), shROPM-1, and shROPM-2 shRNAs (**A**) or non-BCSCs transfected with control lentivirus (LEV) and lncROPM-overexpressed lentivirus (LV-ROPM) (**B**). **C**, **E** Representative images of mammospheres formed from BCSCs transfected with shCtrl, shROPM-1, and shROPM-2 shRNAs (**C**) or non-BCSCs transfected with LEV and LV-ROPM lentivirus (**E**). Original magnification, × 100. Scale bars, 200 µm. **D**, **F** Mammosphere-formation efficiencies in BCSCs transfected with shCtrl, shROPM-1, and shROPM-2 shRNAs (**D**) or non-BCSCs transfected with LEV and LV-ROPM lentivirus (**F**). *n.d.* not detected. **G**, **I** In vitro limiting dilution assay to detect the frequency of stem cells in lncROPM-deficient BCSCs (**G**) or lncROPM-overexpressed non-BCSCs (**I**), as well as their control cells. **H**, **J** LncROPM-silenced (shROPM-2) CSCs (**H**) and lncROPM-overexpressed non-CSCs (**J**) from BT549 were diluted and subcutaneously implanted into BALB/c nude mice, as well as their control cells. Tumors were observed for 2 months. *N* = 5 for each group. Stem cell frequency was calculated by the limiting dilution assay. *CI* confidence interval. Data were presented as the mean ± SD, *p < 0.05, **p < 0.01

To explore the function of lncROPM in BCSCs, lncROPM was knocked down by two of lentivirus-mediated short hairpin RNAs (shRNAs) in BCSCs (Fig. 3A), and ectopic lncROPM was transfected into non-BCSCs with a lentivirus-carried lncROPM (Fig. 3B). Efficient knockdown of lncROPM in BCSCs significantly decreased stemness-related gene (e.g., Sox2, Nanog, Oct4, and Klf4) expressions (Figure S4A-S4B), attenuated sphere sizes (Fig. 3C and Figure S4C-S4D), and reduced the self-renewal capacity in serial passage (Fig. 3D and Additional file 1: Figure S4E), while lncROPM overexpression in non-BCSCs dramatically promoted CSC-related gene expressions (Additional file 1: Figure S5A-S5B), which increased sphere sizes (Fig. 3E and Additional file 1: Figure S5C-S5D) and mammosphere-forming efficiencies in serial passages (Fig. 3F and Additional file 1: Figure S5E). Next, we examined the effect of lncROPM on tumor-initiating potentials by limiting dilution analysis in vitro and in vivo. The results showed that lncROPM deficiency obviously impaired tumor-initiating capacity both in vitro and in vivo (Fig. 3G, H), and ectopic lncROPM could endow the non-CSCs with tumor-initiating ability (Fig. 3I, J). All together, these data suggest that lncROPM contributes to BCSC properties.

### PLA2G16 is a target of lncROPM and promotes breast cancer development and chemo-resistance

**Fig. 4:**
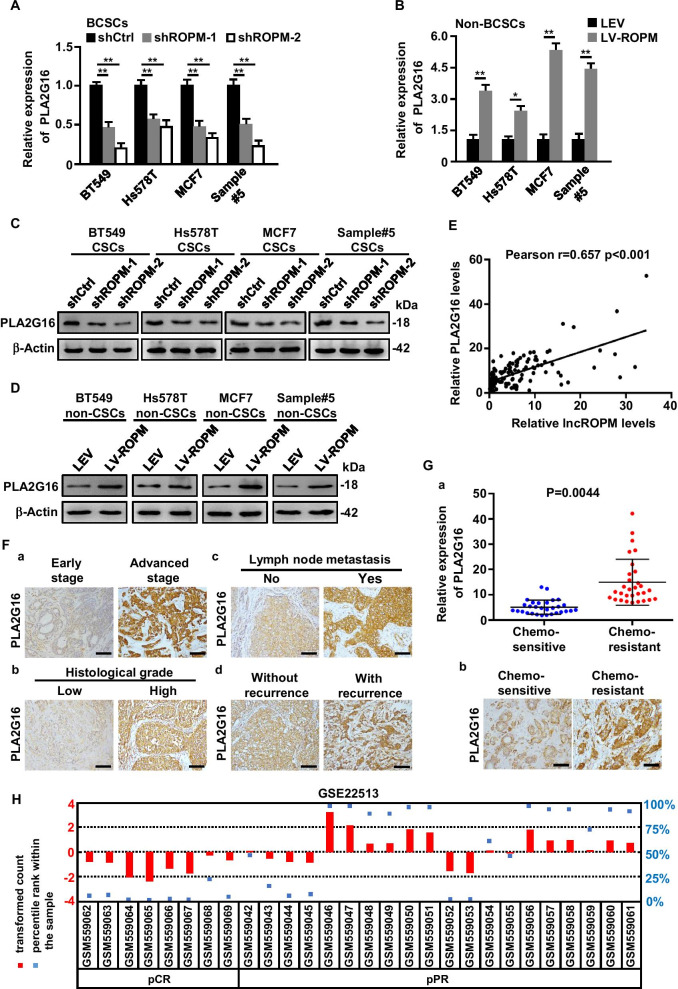
PLA2G16 is a target of lncROPM and associates with breast cancer development and chemo-resistance. **A**, **B** qRT-PCR analysis of PLA2G16 expression in BCSCs transfected with shCtrl or shROPM (**A**), or in non-BCSCs transfected with LEV and LV-ROPM lentivirus (**B**). **C**, **D** Western blotting analysis of PLA2G16 expression in BCSCs or non-BCSC as described in **A** and **B**. **E** Pearson correlation analysis of lncROPM and PLA2G16 expression in 130 breast cancer samples from our data. **F** Representative IHC images of PLA2G16 in breast cancer tissues. Original magnification, × 200. Scale bars, 100 µm. **G** qRT-PCR analysis of PLA2G16 expression in breast cancer chemosensitive tissues (*n* = 30) and chemoresistant tissues (*n* = 30) (left panel). Representative IHC images of PLA2G16 in tissues were shown in the right panel. Original magnification, × 200. Scale bars, 100 µm. **H** PLA2G16 expression in breast tumor samples from patients with pathologic complete response (pCR) and a partial response (pPR) to neoadjuvant paclitaxel/radiation treatment in GEO database (GSE22513). Data were presented as the mean ± SD, *p < 0.05, **p < 0.01

LncRNA was known to play important roles in controlling gene expression. To investigate the underlying mechanisms of lncROPM in regulating BCSCs properties, bioinformatics analysis and qRT-PCR experiments were employed to predict the potential target genes of lncROPM, and we found that the adjacent gene PLA2G16 was a potential target gene for lncROPM (Additional file 1: Figure S6A-S6B). Next, to further test whether lncROPM could influence the expression of PLA2G16, qRT-PCR and western blotting assays were performed, respectively, in different cell lines. As shown, knockdown of lncROPM in BCSCs notably decreased mRNA and protein levels of PLA2G16 (Fig. 4A, C), whereas ectopic lncROPM in non-BCSCs enhanced PLA2G16 levels (Fig. 4B, D) both in breast cancer cell lines and primary breast cancer cells derived from clinic samples. Besides, Pearson correlation analysis indicated a positive correlation between lncROPM and PLA2G16 expressions in our cohort of breast tumors (Fig. 4E), as well as in BCSCs from clinical samples (Additional file 1: Figure S3I). The elevated PLA2G16 levels in breast tumor tissues were closely associated with advanced TNM stage (*p* = 0.004), histology grade (*p* = 0.026), lymph node metastasis (*p* = 0.039), and tumor recurrence (*p* = 0.005) (Table 2). Representative IHC images are shown in Fig. 4F. Similarly, higher levels of PLA2G16 were also observed in chemoresistant breast tumor tissues (Fig. 4G). Analyzed from GEO database, we further found that the enhanced PLA2G16 was in breast cancer tissues with a partial response (pPR) to neoadjuvant paclitaxel/radiation treatment in contrast to these with pathologic complete response (pCR) to neoadjuvant treatment, suggesting that PLA2G16 was closely related to poor prognosis and chemo-resistance in breast cancer patients (Fig. 4H). In shortly, these data support that PLA2G16 is a critical target for lncROPM and contributes to breast cancer development and chemo-resistance.

**Table 2:** Correlation between PLA2G16 expression and clinicopathological features in 130 breast cancer patients

Clinicopathological features	Group	No. of patients, *N* = 130 (%)	*p* value
Age (years)	< 50	72 (55.4)	
	≥ 50	58 (44.6)	*p* = 0.255
Tumor size (cm)	< 3	67 (51.5)	
	≥ 3	63 (48.5)	*p* = 0.078
TNM stage	I–II	70 (53.8)	
	III–IV	60 (46.2)	*p* = 0.004**
Histology grade	I	28 (21.5)	
	II–III	102 (78.5)	*p* = 0.026*
Lymph node metastasis	No	54 (41.5)	
	Yes	76 (58.5)	*p* = 0.039*
Recurrence	No	98 (75.4)	
	Yes	32 (24.6)	*p* = 0.005**

Differences between experimental groups were analyzed by Mann–Whitney test. Data represent mean ± SD (*p < 0.05; **p < 0.01)

### LncROPM regulates PLA2G16 expression by enhancing its mRNA stability

**Fig. 5:**
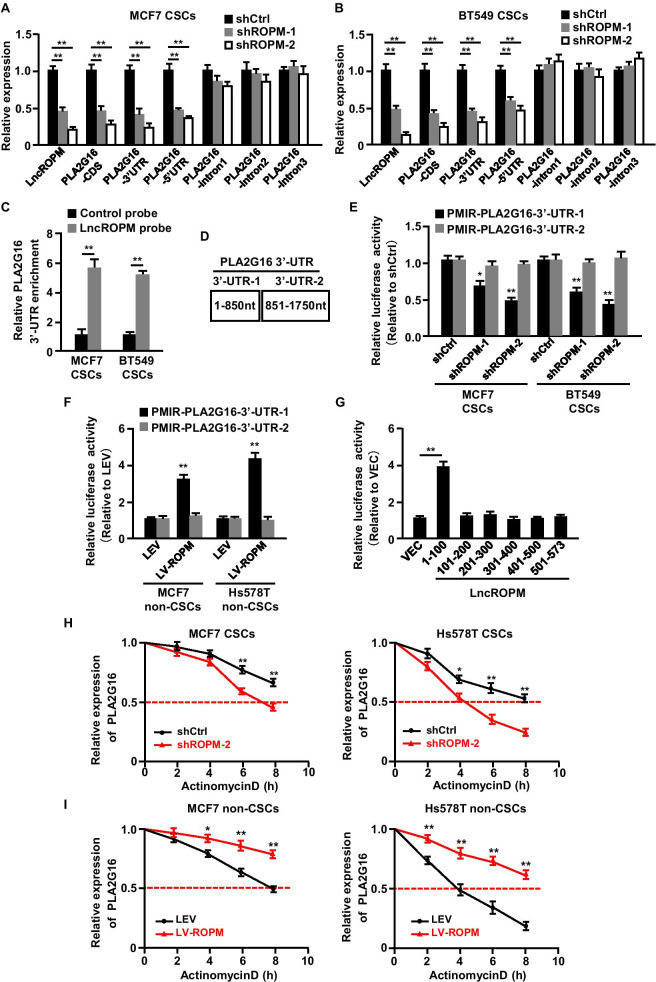
LncROPM binds to the 3'UTR of PLA2G16 mRNA to enhance PLA2G16 mRNA stability. **A**, **B** qRT-PCR analysis of PLA2G16 pre-mRNA (intron1, intron2, intron3) and mature mRNA (3'-UTR, CDS and 5'-UTR) expression in MCF7 BCSCs (**A**) and BT549 CSCs (**B**) transfected with shCtrl, shROPM. **C** RNA pull-down assays were applied to detect the relative enrichment of PLA2G16 3'-UTR in MCF7 and BT549 BCSCs by using the indicated biotinylated probes, and the mRNA level was assessed by using qRT-PCR. **D** PLA2G16 3'-UTR was divided into two parts as shown in diagram. **E**, **F** The luciferase activity of vectors containing different regions of PLA2G16 3'-UTR was determined in BCSCs (**E**) transfected with shCtrl or shROPM, and in non-BCSCs (**F**) with LEV and LV-ROPM. **G** Luciferase activity was measured in 293 T cells co-transfected with lncROPM different fragment plasmid and luciferase reporter containing PLA2G16-3'-UTR-1. **H**, **I** BCSCs with shCtrl or shROPM (**H**) and non-BCSCs with LEV or LV-ROPM (**I**) derived from MCF7 and Hs578T were treated with actinomycin D (2 µg/mL) for the indicated times, respectively. qRT-PCR was used to detect the mRNA half-life of PLA2G16. Data were presented as the mean ± SD, *p < 0.05, **p < 0.01

The mechanisms of lncRNAs largely depend on their intracellular location. Given the cytoplasmic localization of lncROPM, we hypothesized that lncROPM may affect PLA2G16 expression at posttranscriptional level. To verify this hypothesis, we first measured the levels of PLA2G16 pre-mRNA and mature mRNA in lncROPM-deficient BCSCs by qRT-PCR analysis. The results showed that the levels of PLA2G16 mature mRNA (3'-UTR, CDS (coding sequence) and 5'-UTR) were significantly decreased in lncROPM-knockdown BCSCs (Fig. 5A, B, and Additional file 1: Figure S6C). However, the levels of PLA2G16 pre-mRNA containing three intronic regions (intron-1, intron-2, and intron-3) remained non-change, supporting that lncROPM may posttranscriptionally regulate PLA2G16 mRNA expression (Fig. 5A, B, and Additional file 1: Figure S6C). Next, using sequence alignment analysis, we discovered that lncROPM might interact with the 3'-UTR of PLA2G16 mRNA (energy =  − 17.86 kcal/mol) (Additional file 1: Figure S6D-S6E). To test this possibility, we conducted RNA pull-down assays to assess the binding capacity between lncROPM and PLA2G16. We found that there was obvious enrichment of PLA2G16 3'-UTR, but not 5'-UTR or CDS, in the precipitates by lncROPM probe in contrast to control probe (Fig. 5C, and Additional file 1: Figure S6F-S6G). Furthermore, we divided PLA2G16 3'-UTR into two fragments (Fig. 5D). Luciferase reporter assays demonstrated that knockdown or overexpression of lncROPM markedly reduced or enhanced PMIR-PLA2G16-3'-UTR-1 activity, but had no effects on the activities of PMIR-PLA2G16-3'-UTR-2 (Fig. 5E, F). To further determine which fragment of lncROPM is responsible for the interaction with 3'-UTR of PLA2G16, we constructed a set of lncROPM fragment plasmids and co-transfected these constructs with PMIR-PLA2G16-3'-UTR-1 plasmid into 293 T cells, respectively. The results showed that lncROPM fragment (1 to 100 nt) was sufficient to bind with PLA2G16 3'-UTR (Fig. 5G), and the region of lncROPM (37–52 nt) was the most important binding region to the PLA2G16-3'-UTR-1 (Additional file 1: Figure S6H). It has been well known that 3'-UTR is important for mRNA stability. Therefore, we examined the effect of lncROPM on PLA2G16 mRNA stability by using transcriptional inhibitor actinomycin D. We observed that the half-life of PLA2G16 mRNA was rapidly decreased in lncROPM-depleted BCSCs but evidently increased in non-BCSCs with ectopic lncROPM (Fig. 5H, I). Taken together, lncROPM binds to the 3'-UTR of PLA2G16 mRNA to enhance its mRNA stability.

### PLA2G16 is crucial for lncROPM-driven BCSCs properties

**Fig. 6:**
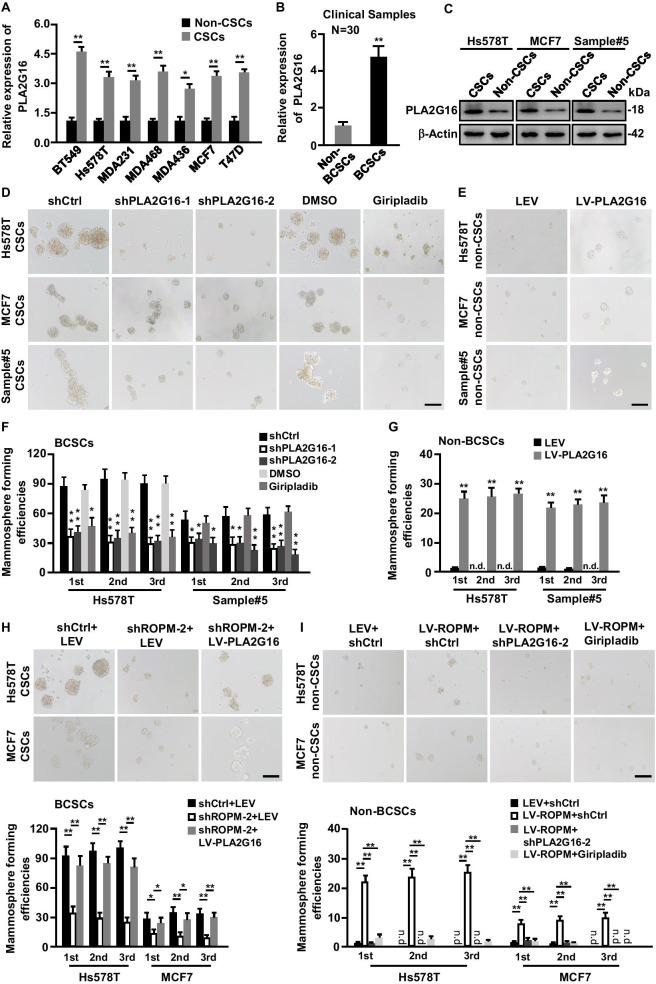
PLA2G16 is crucial for lncROPM-driven BCSCs formation. **A**, **B** qRT-PCR analysis of PLA2G16 expressions in non-BCSCs and BCSCs from various breast cancer cells (**A**) and clinical breast cancer samples (*n* = 30) (**B**). **C** Western blotting to determine PLA2G16 protein levels in non-BCSCs and BCSCs from BT549, MCF7, and clinical sample#5 cells. **D**, **F** Representative mammosphere images (**D**) and mammosphere-formation efficiencies (**F**) of BCSCs transfected with shPLA2G16-1, shPLA2G16-2 or treated with Giripladib (300 nM) for per generation, and their controls. **E**, **G** Representative mammosphere images (**E**) and mammosphere-formation efficiencies (**G**) of non-BCSCs transfected with LEV and LV-PLA2G16 lentivirus. *n.d.* not detected. **H** Ectopic PLA2G16 or control vector was transfected into lncROPM-silenced BCSCs; mammosphere formation abilities were evaluated by suspending culture. The representative mammosphere images (upper panel) and mammosphere-formation efficiencies (down panel) are shown. **I** Mammosphere formation abilities of non-BCSCs with ectopic lncROPM were assessed by suspending culture. And representative mammosphere images (upper panel) and mammosphere-formation efficiencies (down panel) are shown. *n.d.* not detected. Images were taken at the magnification of × 100 and scale bar was 200 µm. Data are presented as the mean ± SD; *p < 0.05, **p < 0.01

To understand whether PLA2G16 regulates BCSC characteristics, we first evaluated the expression of PLA2G16 in BCSCs and non-BCSCs derived from breast cancer cells and clinical tumors by qRT-PCR and western blotting assays. We found that the expression of PLA2G16 was obviously higher in BCSCs than that in non-CSCs (Fig. 6A, C). Moreover, Pearson correlation analysis demonstrated that PLA2G16 was positively associated with CSC-related gene (e.g., Sox2 and Oct4) expression in breast tumor tissues (Additional file 1: Figure S7A-S7B). Then, we established stable PLA2G16-deficient BCSCs using two different specific lentivirus-mediated shRNAs, as well as stable PLA2G16-overexpressed non-BCSCs by a lentivirus-carried PLA2G16 (Additional file 1: Figure S7C-S7F). Meanwhile, Giripladib, an inhibitor of cytoplasmic phospholipase A2 including PLA2G16 enzyme, which was predominantly located in the cytoplasm of BCSCs (Additional file 1: Figure S7G), was also used. Efficient knockdown of PLA2G16 or using Giripladib in BCSCs remarkably reduced stemness-related gene (e.g., Sox2, Oct4, Klf4) expression (Additional file 1: Figure S8A), and decreased mammosphere forming efficiencies in serial passages (Fig. 6D, F) and sphere sizes of BCSCs (Additional file 1: Figure S8B), whereas PLA2G16 overexpression in non-BCSCs promoted stemness phenotypes (Fig. 6E, G; and Additional file 1: Figure S8C-S8D). These data indicate that PLA2G16 contributes to BCSCs stemness features.

To further confirm the role of PLA2G16 in CSCs, PLA2G16 highly expressed (PLA2G16^+/high^) and PLA2G16 lowly expressed (PLA2G16^−/low^) cells were isolated from breast cancer cells by flow cytometry assay. We observed that CD44^+^/CD24^−/low^ cells population and CSC-related genes were significantly increased in PLA2G16^+/high^ cells compared with PLA2G16^−/low^ cells (Additional file 1: Figure S9A-S9B). More interestingly, analyzed from GEO database, we further found that PLA2G16 was highly expressed in embryonic stem cells than its progeny cells and embryoid bodies (Additional file 1: Figure S9C-S9D). Similarly, in the liver, PLA2G16 expression was much higher in resident stem cells than that in differentiated cells (Additional file 1: Figure S9E). Besides, PLA2G16 was also upregulated in response to the stimulation of stem cell factor (SCF) in bone marrow-derived mast cells (Additional file 1: Figure S9F), implying that PLA2G16 may promote stem cell self-renewal and proliferation. Taken together, these findings suggest that PLA2G16 involves in regulating CSC stemness properties and may serve as a biomarker for BCSCs and even other stem cells.

Furthermore, to explore whether lncROPM regulates BCSCs stemness characteristics in a PLA2G16-dependent manner, we conducted rescue experiments. Our results demonstrated that ectopic PLA2G16 could partially rescue stemness-related genes expressions (Additional file 1: Figure S10A-S10B) and self-renewal capacity in serial passages (Fig. 6H) caused by lose of lncROPM in BCSCs, while these enhanced CSC phenotypes in non-BCSCs induced by lncROPM overexpression were obviously reversed by PLA2G16 silencing or inactivation (Additional file 1: Figure S10C-S10D, and Fig. 6I). These results demonstrate the function of lncROPM on BCSCs via upregulating PLA2G16.

### LncROPM promotes BCSCs stemness by regulating PLA2G16-mediated phospholipid metabolism

**Fig. 7:**
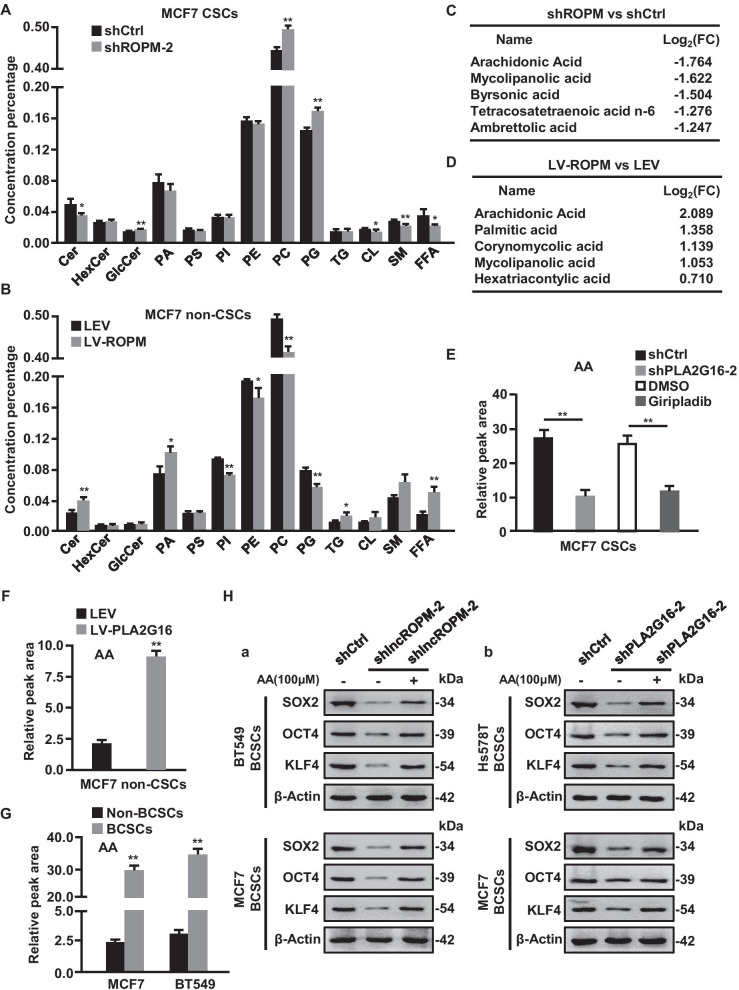
PLA2G16-mediated phospholipid metabolism and metabolite arachid-onic acid (AA) involve in LncROPM-driven BCSCs maintenance. **A**, **B** The altered lipid species in MCF7 BCSCs (**A**) with or without lncROPM, or MCF7 non-BCSCs (**B**) with or without ectopic lncROPM. *Cer* ceramide; *HexCer* hexosylceramide; *GlcCer* glucosylceramide; *PA* phosphatidic acid; *PS* phosphatidylserine; *PI* phosphatidylinositol; *PE* phosphatidylethanolamine; *PC* phosphatidylcholine; *PG* glycerophosphoglycerols; *TG* triglyceride; *CL* cardiolipin; *SM* sphingomyelin; *FFA* free fatty acid. **C**, **D** The top 5 changed FFAs in lncROPM-silenced MCF7 BCSCs group (**C**) or ectopic lncROPM-expressing MCF7 non-BCSCs group (**D**). **E** PLA2G16 in MCF-7 BCSCs were knocked down by shRNA or pre-treated with Giripladib (300 nM); the contents of AA were measured in these BCSCs and their control BCSCs. **F**, **G** The AA contents in MCF7 non-CSCs transfected with or without LV-ROPM (**F**), and in non-BCSCs and BCSCs derived from MCF7 and BT549 cells. **H** Western blotting to determine stemness-related proteins (SOX2, OCT4, and KLF4) in shCtrl group, shlncROPM-2 group treated with or without AA (100 µM) from BT549 BCSCs and MCF7 BCSCs for 7 days (**a**) or shCtrl group, shPLA2G16-2 group treated with or without AA (100 µM) from Hs578T BCSCs and MCF7 BCSCs for 7 days (**b**). Data are presented as the mean ± SD; *p < 0.05, **p < 0.01

It has been reported that PLA2G16 involved in the process of phospholipid metabolism [22, 39]. To prove whether lncROPM affects BCSC characteristics through the alteration of phospholipid metabolism induced by PLA2G16, and which metabolites play a pivotal role to BCSC stemness, lipidomics of lncROPM-knocked down BCSCs and lncROPM-overexpressing non-BCSCs were carried out using UHPLC-QTOFMS system. We identified PC (phosphatidylcholines) and PG (glycerophosphoglycerols), two of metabolic substrates of PLA2G16, were significantly increased in lncROPM-knocked down BCSC and decreased in lncROPM-overexpressing non-BCSCs, whereas Cer (Ceramides) and FFA (free fatty acids), two of representative metabolites of PLA2G16, dramatically reduced in lncROPM-silenced BCSCs and elevated in lncROPM overexpressing non-BCSC (Fig. 7A, B), suggesting that Cer and FFA might be the key metabolites associated with lncROPM-PLA2G16-driven BCSC properties.

In order to deeply study the impact of metabolites on BCSC properties, we focused on FFA rather than Cer for further investigation, due to its highest change in this study. Among the top five changed metabolites of FFA, arachidonic acid (AA) was the most significantly changed metabolite (Fig. 7C, D). To confirm AA was closely related to PLA2G16, we measured AA contents in PLA2G16-knockdown or Giripladib-treated BCSCs as well as PLA2G16-overexpressing non-BCSCs and found that AA was regulated by PLA2G16 (Fig. 7E, F). Meanwhile, AA was markedly enriched in BCSCs compared with non-BCSCs (Fig. 7G). Besides, there were notable high contents of AA in advanced-stage tumor tissues, recurrent tumor tissues, and chemoresistant tumors (Additional file 1: Figure S11A-S11C). These evidences indicate that AA is crucial for maintenance of BCSCs and may work as a biomarker of breast cancer malignancy in clinic.

To further validate its function of AA on BCSCs, exogenous AA was applied in BCSCs property analysis. AA treatment could rescue the repressed stemness-related gene (e.g. Sox2, Oct4, and Klf4) expression and mammosphere-forming efficiency caused by sh-lncROPM or sh-PLA2G16 in BCSCs (Fig. 7H, and Additional file 1: Figure S11D-11E). Subsequently, we evaluated the classical stem cell-related signaling (e.g., Wnt/β-catenin, Notch, Hedgehog, Hippo/YAP, PI3K/AKT) activities and found that AA could specially activate PI3K/AKT, Wnt/β-catenin, and Hippo/YAP signaling (Additional file 1: Figure S11F-11G). Taken together, these data confirm that lncROPM-PLA2G16 signal axis regulates phospholipid metabolism leading an enhanced AA product, thereby activating PI3K/AKT, Wnt/β-catenin, and Hippo/YAP signaling to promote BCSCs stemness.

### Giripladib combining with chemotherapeutic drugs can efficiently eliminate BCSCs

**Fig. 8:**
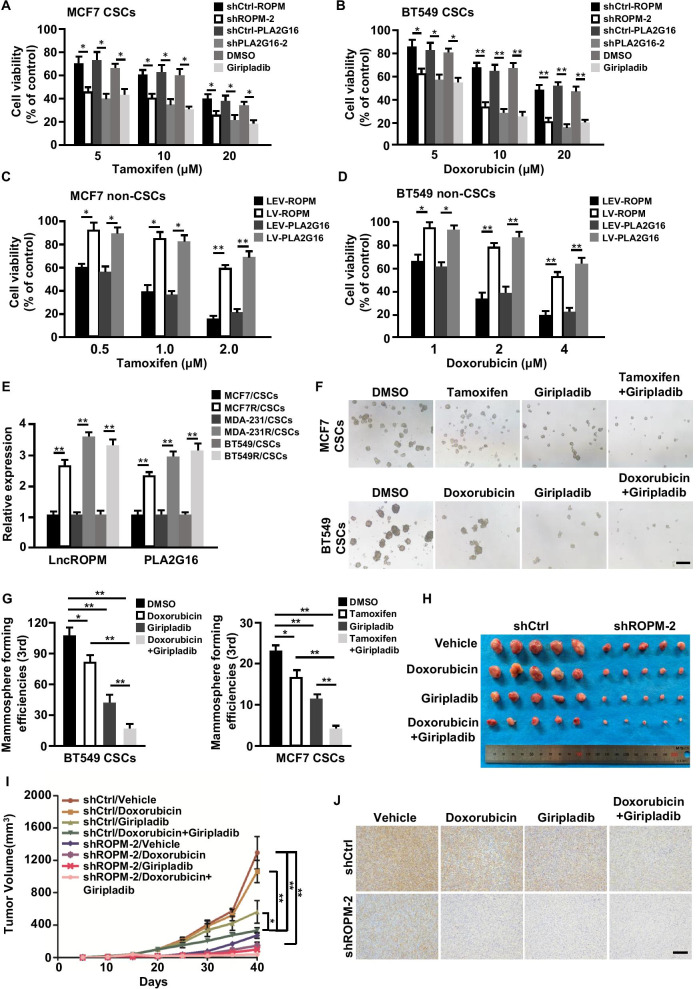
The treatment of Giripladib combined with clinic therapeutic drugs efficiently eliminates BCSCs. **A**–**D** The indicated BCSC or non-BCSC cells were treated with designed tamoxifen (**A**, **C**) or doxorubicin (**B**, **D**) for 48 h. Cell viabilities were measured by CCK-8 assay. **E** qRT-PCR analysis of lncROPM and PLA2G16 expressions in BCSCs from breast cancer resistant cells and their parental cells. **F**, **G** Representative images of mammospheres (**F**) and mammosphere-formation efficiencies (**G**) of BCSCs from MCF7 treated with DMSO, Tamoxifen, Giripladib, Tamoxifen + Giripladib or Hs578T treated with DMSO, Doxorubicin, Giripladib, or Doxorubicin + Giripladib. Original magnification, × 40. Scale bars, 400 μm. **H** Representative images of xenograft tumors for each group (*n* = 5). **I** Xenograft tumors volume. **J** Representative images of IHC staining of KLF4 in each of xenograft tumor group (magnification, × 200. Scale bar, 100 µm). Data are presented as the mean ± SD; *p < 0.05, **p < 0.01

In the aforementioned data, we found that there were high levels of lncROPM and PLA2G16 in chemotherapy-resistant breast cancer tissues (Fig. 2K and 4G). To further verify the role of lncROPM and PLA2G16 on drug resistance of breast cancer, the effects of lncROPM and PLA2G16 on BCSCs survival in response to chemotherapeutic drugs were investigated. Our data showed that knockdown of lncROPM or PLA2G16 increased BCSCs sensitivity to chemotherapeutic drugs (tamoxifen, doxorubicin, cisplatin) (Fig. 8A, B, and Additional file 1: Figure S12A). Of note, treating BCSCs with Giripladib had similar results (Fig. 8A, B, and Additional file 1: Figure S12A). However, exogenous addition of AA to lncROPM- or PLA2G16-depleted BCSCs, or Giripladib-treated BCSCs could rescue their drug sensitivity (Additional file 1: Figure S12B-S12D). Moreover, overexpression of lncROPM or PLA2G16 in non-BCSCs decreased its sensitivity to chemotherapeutic drugs (Fig. 8C, D, and Additional file 1: Figure S12E). Next, we examined lncROPM and PLA2G16 expressions in BCSCs from tamoxifen-resistant MCF-7 cells, cisplatin-resistant MDA-MB-231 cells, doxorubicin-resistant BT549 cells and their parental cells. As expected, the enhanced lncROPM and PLA2G16 were detected in these drug-resistant BCSCs, which possessed strong stemness properties in comparison with parental BCSCs (Fig. 8E, Additional file 1: Figure S12F), indicating that lncROPM, PLA2G16, and AA endow BCSCs with chemoresistant ability, while Giripladib can suppress drug resistance of BCSC by inhibiting PLA2G16 enzyme activity. Therefore, we tried to employ Giripladib in combination with clinic drugs (doxorubicin, cisplatin, or tamoxifen) to eliminate BCSCs. Fortunately, Giripladib combined with clinic drugs had more effective in reducing BCSCs numbers than a single drug treatment alone (Fig. 8F, G, and Additional file 1: Figure S12G-S12J) checked by mammosphere-forming assays. Subsequently, we want to validate these findings in vivo using nude mice xenograft model. Consistently, compared with the monotherapy group, Giripladib combined with chemotherapeutic drug (doxorubicin) notably decreased tumorigenesis of BCSCs and suppressed tumor growth (Fig. 8H, I). Correspondingly, stemness-related transcription factor KLF4, one of representative BCSC protein, was significantly reduced in the tumors accepted combination treatment (Fig. 8J) by immunohistochemistry (IHC) staining. Taken together, these data demonstrate that lncROPM, PLA2G16, and AA contribute to BCSCs drug resistance, and Giripladib synergizing with chemotherapeutic drugs can efficiently eliminate BCSCs by suppressing PLA2G16 activity.

## Discussion

Cancer stem cells (CSCs) are considered to be the major drivers of tumor growth, metastasis, recurrence, and heterogeneity [40], and lead to poor outcomes in patients [41]. In recent years, emerging evidence has shown that metabolic reprogramming played a pivotal role in controlling CSCs fate [11, 42, 43]. However, the role and function of many potential metabolism-related genes in CSCs are still not fully elucidated, such as circRNAs, lncRNAs, microRNAs, and even some mRNAs. To date, with the rapid development of RNA genomics, more and more oncogenic and suppressive lncRNAs have been annotated in various tumors, indicating that lncRNAs have great biological significance in tumor development and may become beneficial tumor biomarkers for clinical diagnosis and therapy [44, 45]. In our study, we defined a novel metabolism-related lncRNA named lncROPM, which is highly expressed in BCSCs and involves in BCSCs renewal, stemness, and drug resistance by regulating phospholipid metabolism through targeting the expression of PLA2G16.

More importantly, we explored the expression of lncROPM in clinical specimens from breast cancer patients and TCGA-BRCA database (RNAseq data). It was intriguing that lncROPM is significantly associated with tumor malignancy, recurrence, chemo-resistance, and poor prognosis in patients with breast cancer. These findings suggest that lncROPM can serve as a new diagnostic and prognostic marker as well as therapeutic target for breast cancer patients. Besides, we also found that lncROPM is closely correlated with poor prognosis of patients with kidney renal clear cell carcinoma (KIRC) and strongly contributed to head and neck squamous cell carcinoma (HNSC) and ovarian serous cystadenocarcinoma (OV) development basing on TANRIC database analysis, implying that lncROPM might act as an oncogene in tumors. But the specific function and regulatory mechanism of lncROPM in other tumors need to be further validated.

Recently, lncRNAs are frequently reported to be co-expressed with chromosomally adjacent genes through cis-regulatory mechanisms [46–48]. Herein we identified PLA2G16, a neighboring coding-gene, as a target for lncROPM. Furthermore, PLA2G16 is positively associated with the expression of lncROPM both in BCSCs and clinical specimens. It has been reported that lncRNAs are widely expressed in multiple cellular fractions, like nucleus, nucleolus, cytoplasm, and even the mitochondria [49]. The localization of lncRNAs is a great indicator for their mode of function [49, 50]. In this study, RNA-FISH and subcellular fractionation tests showed that lncROPM is mainly distributed in the cytoplasm of BCSCs, prompting that lncROPM probably affects the expression of PLA2G16 via modulating polysome fractions, controlling mRNA stability, or participating in translation-related activities. Subsequently, our results proved that lncROPM can potentiate PLA2G16 mRNA stability by binding to its 3’-UTR region. Generally, lncRNAs can influence mRNAs stability in several ways, such as acting as miRNA sponges to compete for miRNA binding and then protect miRNA targets from degradation, recruiting proteins to modulate mRNA stability, or serving as molecular decoys for RBPs to regulate mRNA decay [17]. However, it attracted our attention that unlike the common patterns we observed that lncRNA can facilitate mRNA stability via directly binding to its 3’-UTR region. Nonetheless, there are also a few similar researches supporting our views; for instance, lncRNA THOR can directly bind to SOX9 3’-UTR, instead of 5’-UTR or coding area to enhance its mRNA stability, thereby increasing SOX9 expression [51]. In a word, our study broadens the knowledge of regulatory mechanisms in lncRNAs.

Initially, PLA2G16 was defined as a tumor suppressor due to the inhibitory impact on H-Ras [18]. But in contrast, increasing studies indicate that PLA2G16 has oncogenic activity in tumors, including non-small cell lung cancer, osteosarcoma, colorectal cancer, gastric cancer, and rectum cancer [21, 27–30]. As for breast cancer, until now only a few of papers have reported about PLA2G16. One study illustrated that PLA2G16 overexpression contributes to metastatic potential of mammary tumors [29], and the other demonstrated that high expression of PLA2G16 is correlated with better prognosis in HER2-positive patients [52]. However, the specimens collected in the latter research were mainly from early-stage patients (70 in Stage I, 23 in Stage II, and 1 not available), and the expression of PLA2G16 was only quantified by IHC experiments, making the conclusion not convincing enough. In addition, both of the two articles did not investigate how PLA2G16 affects the development of breast cancer. Strikingly, PLA2G16 can also function as a phospholipase, which generates free fatty acids, particularly arachidonic acid from phospholipids [22]. Nevertheless, whether PLA2G16 can promote tumor progression by impacting phospholipid metabolism in tumors remains unknown. Moreover, there is also no literature surrounding the influence of PLA2G16 on CSCs.

Here, we first validated that PLA2G16 is overexpressed in BCSCs and obviously contributes to its self-renewal capacity, stem cell markers expression, and drug resistance. Besides, the meta-analysis from GEO database displayed that PLA2G16 is also highly expressed in musculus embryonic stem cells, resident liver stem cells, and homo sapiens undifferentiated embryonic stem cells. These data broaden the scope of the study and provide additional insights into the effect of PLA2G16 on stem cells, implying that PLA2G16 may be used as a new molecular marker to define BCSCs, even the stem cells in other tissues. Of note, in our study, clinical studies showed that the expression of PLA2G16 is markedly related to breast cancer malignancy, recurrence, chemo-resistance and poor prognosis in patients, suggesting that PLA2G16 is a useful therapeutic target to eradicate BCSCs and improve the poor prognosis of patients with breast cancer. Excitingly, we found that the cytoplasmic PLA2 inhibitor Giripladib can potently reduce BCSCs stemness and chemo-resistance. The combination of Giripladib and chemotherapy drug doxorubicin can kill BCSCs both in vivo and in vitro.

To date, researches are predominantly focused on the relationship between key metabolic enzymes and disease pathogenesis [53, 54]. The effect of altered metabolites mediated by enzymes on disease development has yet been well understood. In this study, we observed that the content of arachidonic acid (AA) is significantly changed in BCSCs. It is universally known that AA is a polyunsaturated ω-6 fatty acid present in the phospholipids and considered as a critical mediator of cancer signaling transduction involving in various biological processes, such as proliferation, migration, invasion, and chemo-resistance [24, 55, 56]. However, there are few publications on the role of AA in CSCs. Herein, we found that AA is higher in BCSCs than non-CSCs and promotes BCSCs self-renewal, stemness, chemo-resistance via activating PI3K/AKT, Wnt/β-catenin, and Hippo/YAP signaling pathways. On the other hand, activated PI3K signaling was reported to regulate arachidonic acid metabolism [57]. This suggested that PI3K signaling and arachidonic acid may form a positive feedback loop to further promote the malignant behavior in some canner cells. However, this hypothesis needs to be more evidence in the future studies.

## Conclusions

In conclusion, our study highlights the importance of lncROPM and its target PLA2G16 in modulating BCSCs, thereby promoting breast cancer development, recurrence, and chemo-resistance. These results suggest that lncROPM and PLA2G16 are a useful biomarker to identify and treat BCSCs in breast cancer patients. Particularly, we also provide a novel clinical treatment strategy for breast cancer patients.

## Supplementary Information

**Additional file 1.**. Supplementary figures.

**Additional file 2.** Oligomers used in this study.

## Data Availability

The supporting data and materials are provided in Additional Figures and Tables.

## Abbreviations

LncRNA: Long noncoding RNA
CSCs: Cancer stem cells
BCSCs: Breast cancer stem cells
LncROPM: A regulator of phospholipid metabolism
PLA2G16: Group XVI phospholipase A2
FISH: Fluorescence in situ hybridization
TCGA: The Cancer Genome Atlas
BRCA: Breast invasive carcinoma
KIRC: Kidney renal clear cell carcinoma
HNSCC: Head and neck squamous cell carcinoma
OV: Ovarian serous cystadenocarcinoma
qRT-PCR: Quantitative reverse transcription polymerase chain reaction
shRNA: Short hairpin RNA
IHC: Immunohistochemistry
3’-UTR: 3′-Untranslated region
5'-UTR: 5′-Untranslated region
CDS: Coding sequence
GEO: Gene expression omnibus
FFA: Free fatty acids
AA: Arachidonic acid
