# Supplementary material for: A novel lncRNA ROPM-mediated lipid metabolism governs breast cancer stem cell properties
Source: J Hematol Oncol. 2021 Oct 29;14:178. doi: 10.1186/s13045-021-01194-z (PMC8555326; doi:10.1186/s13045-021-01194-z)
Supplement: Supplementary file 1 — Additional file 1.. Supplementary figures. [file 13045_2021_1194_MOESM1_ESM.pptx]

## Slide 1
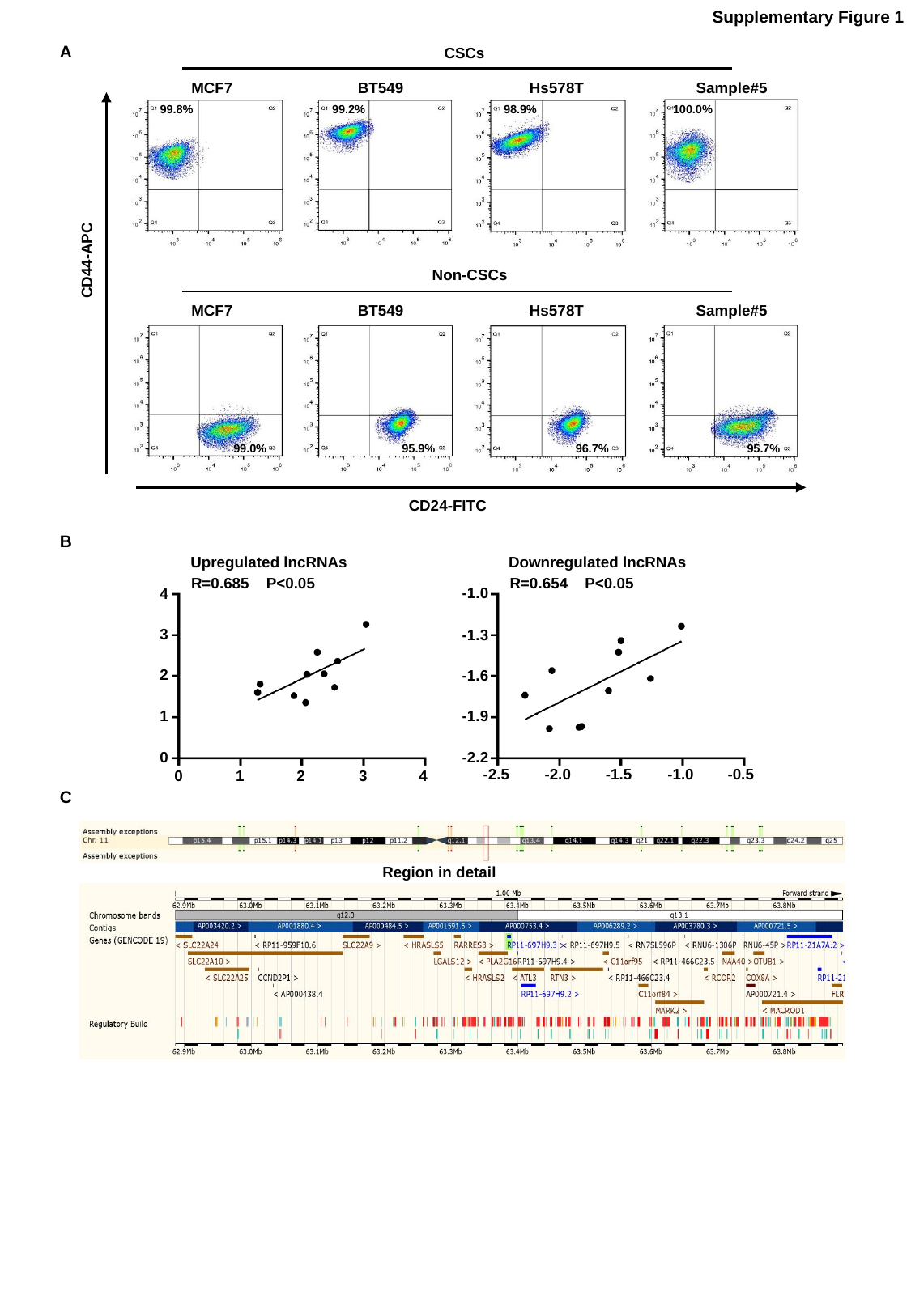

Supplementary Figure 1
A
CSCs
MCF7
BT549
Hs578T
Sample#5
99.8%
99.2%
98.9%
100.0%
CD44-APC
Non-CSCs
MCF7
BT549
Hs578T
Sample#5
99.0%
95.9%
96.7%
95.7%
CD24-FITC
B
Upregulated lncRNAs
Downregulated lncRNAs
R=0.685 P<0.05
R=0.654 P<0.05
-1.0
-1.3
-1.6
-1.9
-2.2
-2.5
-2.0
-1.5
-1.0
-0.5
4
3
2
1
0
0
1
2
3
4
C
Region in detail

## Slide 2
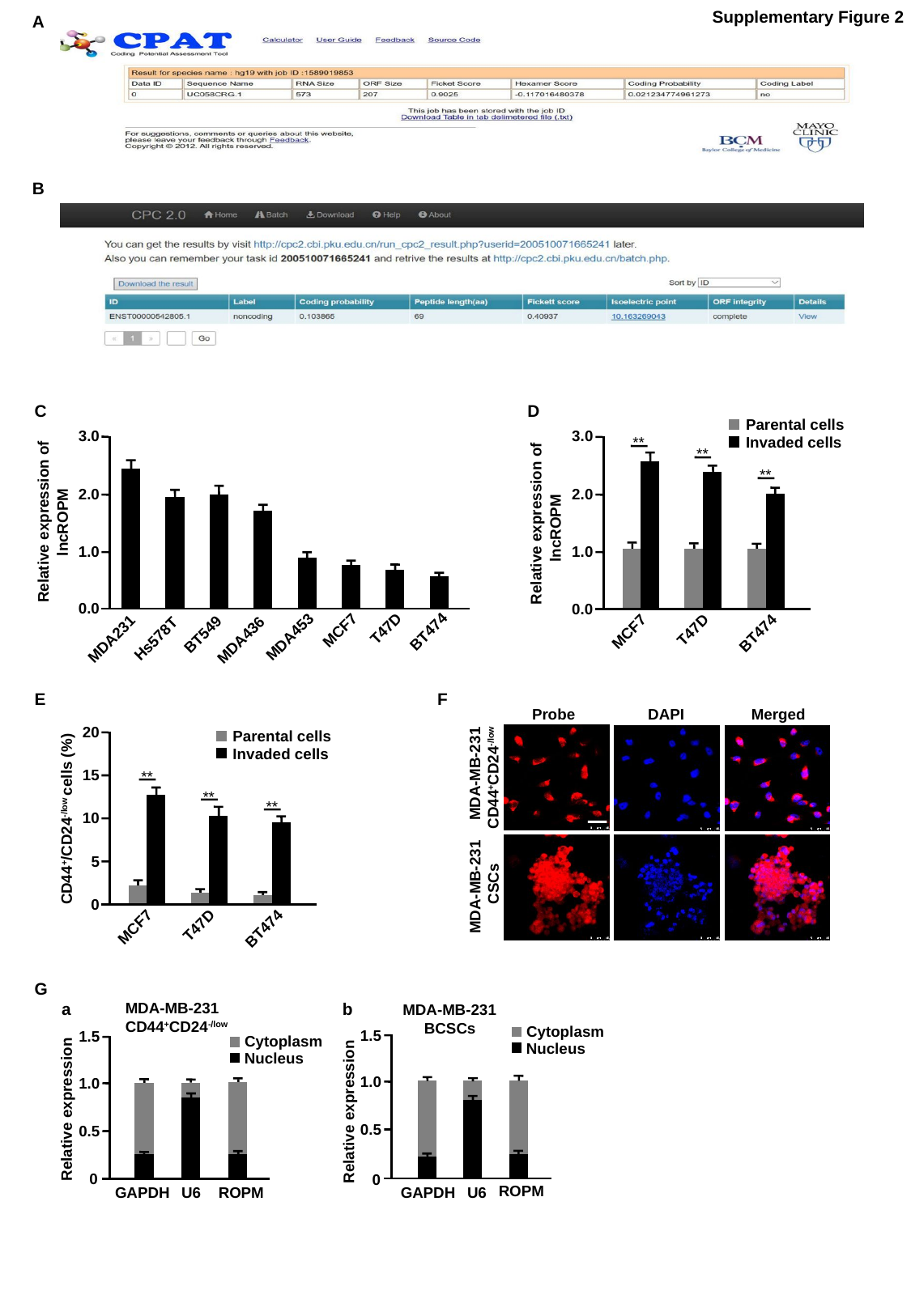

Supplementary Figure 2
A
B
C
D
Parental cells
Invaded cells
3.0
**
**
**
2.0
 Relative expression of
 lncROPM
1.0
0.0
T47D
MCF7
BT474
3.0
2.0
 Relative expression of
 lncROPM
1.0
0.0
T47D
BT474
MCF7
BT549
MDA453
MDA231
Hs578T
MDA436
E
F
Probe
DAPI
Merged
 MDA-MB-231
CD44+CD24-/low
MDA-MB-231
 CSCs
20
Parental cells
Invaded cells
15
**
**
**
10
CD44+/CD24-/low cells (%)
5
0
T47D
MCF7
BT474
G
a
b
MDA-MB-231
CD44+CD24-/low
Cytoplasm
Nucleus
1.5
1.0
 Relative expression
0.5
0
GAPDH
U6
ROPM
MDA-MB-231
 BCSCs
Cytoplasm
Nucleus
1.5
1.0
 Relative expression
0.5
0
ROPM
GAPDH
U6

## Slide 3
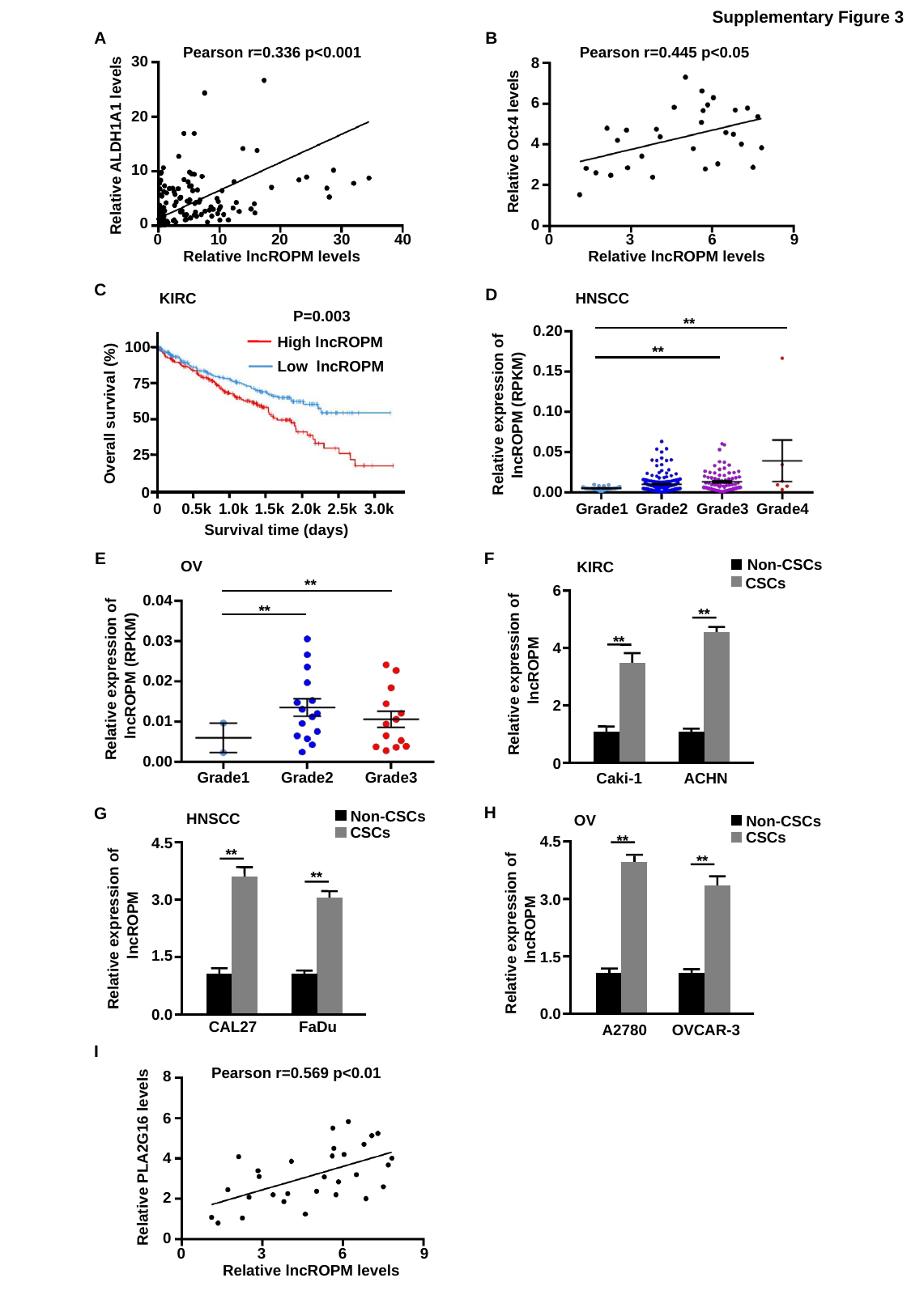

Supplementary Figure 3
A
B
Pearson r=0.336 p<0.001
30
20
Relative ALDH1A1 levels
10
0
0
10
20
30
40
Relative lncROPM levels
Pearson r=0.445 p<0.05
8
6
Relative Oct4 levels
4
2
0
0
3
6
9
Relative lncROPM levels
C
D
KIRC
P=0.003
High lncROPM
Low lncROPM
100
75
Overall survival (%)
50
25
0
0
0.5k
1.0k
1.5k
2.0k
2.5k
3.0k
Survival time (days)
HNSCC
**
**
0.20
0.15
Relative expression of
 lncROPM (RPKM)
0.10
0.05
0.00
Grade1
Grade2
Grade3
Grade4
F
E
Non-CSCs
CSCs
KIRC
6
**
**
4
Relative expression of
 lncROPM
2
0
Caki-1
ACHN
OV
**
**
0.04
0.03
Relative expression of
 lncROPM (RPKM)
0.02
0.01
0.00
Grade1
Grade2
Grade3
H
G
Non-CSCs
CSCs
HNSCC
4.5
**
**
3.0
Relative expression of
 lncROPM
1.5
0.0
CAL27
FaDu
OV
Non-CSCs
CSCs
**
4.5
**
3.0
Relative expression of
 lncROPM
1.5
0.0
A2780
OVCAR-3
I
Pearson r=0.569 p<0.01
8
6
Relative PLA2G16 levels
4
2
0
0
3
6
9
Relative lncROPM levels

## Slide 4
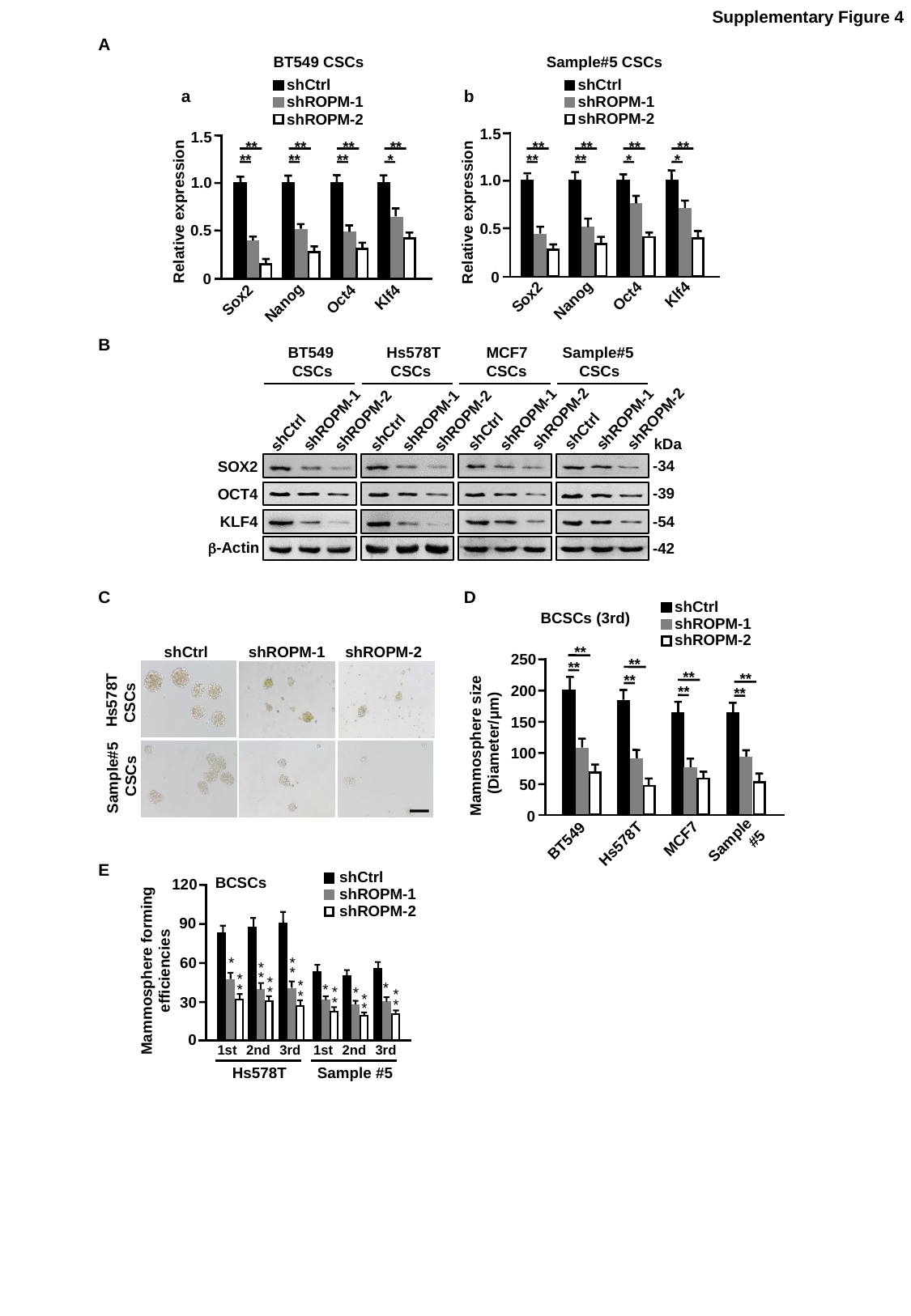

Supplementary Figure 4
A
BT549 CSCs
Sample#5 CSCs
shCtrl
shROPM-1
shROPM-2
shCtrl
shROPM-1
shROPM-2
a
b
1.5
1.5
**
**
**
**
**
**
**
**
**
**
**
**
**
*
*
*
1.0
1.0
 Relative expression
 Relative expression
0.5
0.5
0
0
Klf4
Oct4
Sox2
Klf4
Oct4
Sox2
Nanog
Nanog
B
BT549
 CSCs
Hs578T
 CSCs
MCF7
CSCs
Sample#5
 CSCs
shROPM-2
shROPM-1
shROPM-1
shROPM-1
shROPM-1
shROPM-2
shROPM-2
shROPM-2
shCtrl
shCtrl
shCtrl
shCtrl
kDa
-34
SOX2
-39
OCT4
KLF4
-54
b-Actin
-42
C
D
shCtrl
shROPM-1
shROPM-2
BCSCs (3rd)
**
**
250
**
**
**
**
**
**
200
Mammosphere size
 (Diameter/μm)
150
100
50
0
Sample
 #5
BT549
MCF7
Hs578T
shCtrl
shROPM-1
shROPM-2
Hs578T
 CSCs
Sample#5
 CSCs
E
shCtrl
shROPM-1
shROPM-2
BCSCs
120
90
Mammosphere forming
 efficiencies
60
*
*
*
*
*
*
*
*
*
*
*
*
*
*
*
*
*
*
30
*
*
0
1st
2nd
3rd
1st
2nd
3rd
Hs578T
Sample #5

## Slide 5
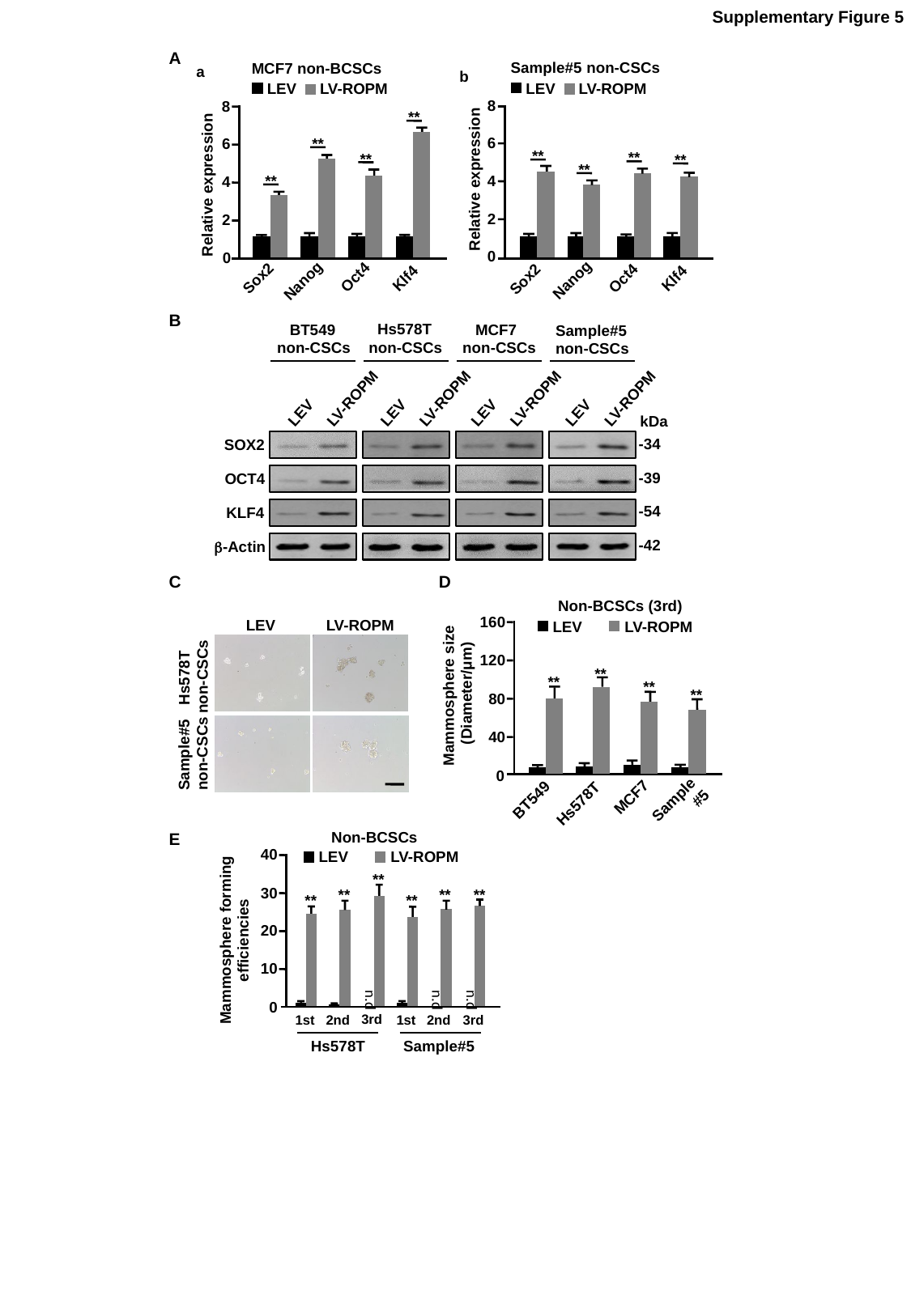

Supplementary Figure 5
A
Sample#5 non-CSCs
MCF7 non-BCSCs
a
LEV
LV-ROPM
8
**
**
6
**
**
4
 Relative expression
2
0
Klf4
Oct4
Sox2
Nanog
b
LEV
LV-ROPM
8
6
**
**
**
**
4
 Relative expression
2
0
Klf4
Oct4
Sox2
Nanog
B
 Hs578T
non-CSCs
 BT549
 non-CSCs
 MCF7
non-CSCs
Sample#5
non-CSCs
LV-ROPM
LV-ROPM
LV-ROPM
LV-ROPM
LEV
LEV
LEV
LEV
kDa
-34
SOX2
-39
OCT4
-54
KLF4
-42
b-Actin
C
D
 Non-BCSCs (3rd)
LEV
LV-ROPM
160
120
**
**
Mammosphere size
 (Diameter/μm)
**
**
80
40
0
Sample
 #5
MCF7
BT549
Hs578T
LEV
LV-ROPM
 Hs578T
non-CSCs
Sample#5
non-CSCs
Non-BCSCs
LEV
LV-ROPM
40
**
**
**
**
30
**
**
Mammosphere forming
 efficiencies
20
10
n.d
n.d
n.d
0
3rd
1st
2nd
1st
2nd
3rd
Hs578T
Sample#5
E

## Slide 6
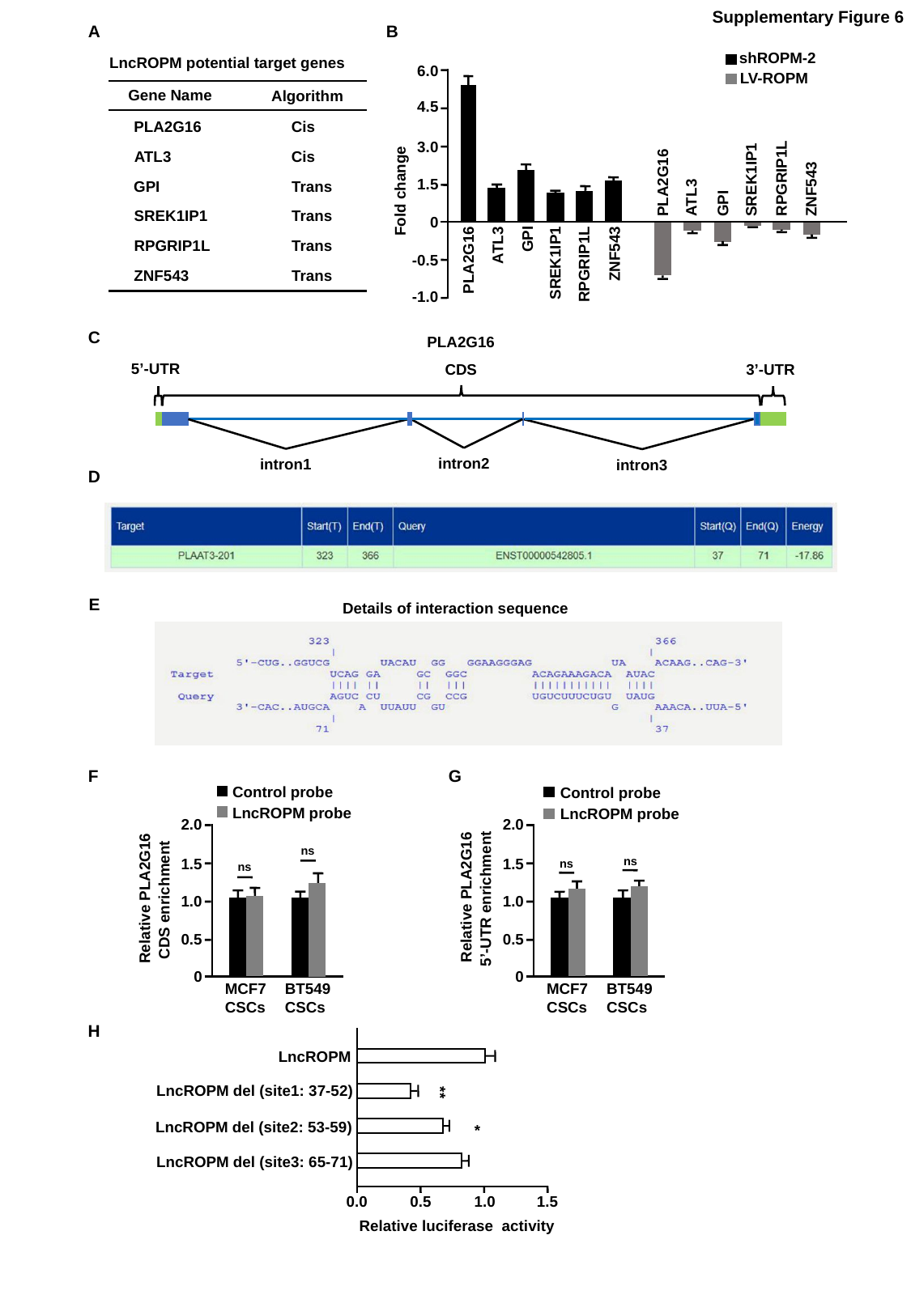

Supplementary Figure 6
A
B
shROPM-2
LV-ROPM
LncROPM potential target genes
Gene Name
Algorithm
PLA2G16
Cis
ATL3
Cis
GPI
Trans
SREK1IP1
Trans
RPGRIP1L
Trans
ZNF543
Trans
6.0
4.5
3.0
RPGRIP1L
SREK1IP1
PLA2G16
1.5
ZNF543
Fold change
ATL3
GPI
0
GPI
ATL3
ZNF543
-0.5
PLA2G16
SREK1IP1
RPGRIP1L
-1.0
C
PLA2G16
5’-UTR
CDS
3’-UTR
intron2
intron1
intron3
D
E
Details of interaction sequence
F
Control probe
LncROPM probe
2.0
ns
1.5
ns
 Relative PLA2G16
 CDS enrichment
1.0
0.5
0
MCF7
CSCs
BT549
CSCs
G
Control probe
LncROPM probe
2.0
ns
1.5
ns
 Relative PLA2G16
 5’-UTR enrichment
1.0
0.5
0
MCF7
CSCs
BT549
CSCs
H
LncROPM
LncROPM del (site1: 37-52)
**
LncROPM del (site2: 53-59)
*
LncROPM del (site3: 65-71)
0.0
0.5
1.0
1.5
Relative luciferase activity

## Slide 7
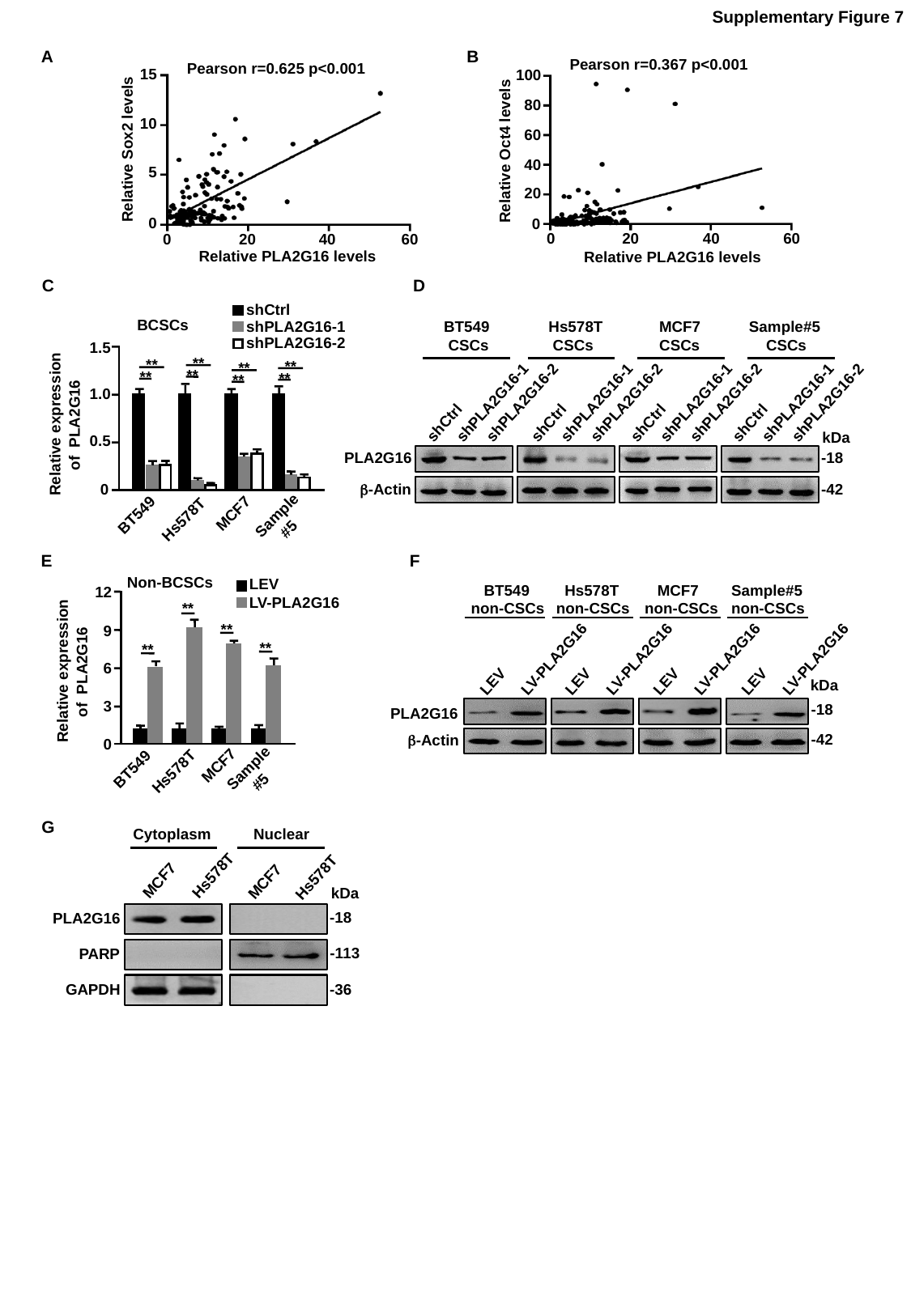

Supplementary Figure 7
A
B
Pearson r=0.625 p<0.001
15
10
Relative Sox2 levels
5
0
0
20
40
60
Relative PLA2G16 levels
Pearson r=0.367 p<0.001
100
80
60
Relative Oct4 levels
40
20
0
0
20
40
60
Relative PLA2G16 levels
C
D
shCtrl
shPLA2G16-1
shPLA2G16-2
BCSCs
1.5
**
**
**
**
**
**
**
**
1.0
 Relative expression
 of PLA2G16
0.5
0
Sample
 #5
BT549
MCF7
Hs578T
BT549
 CSCs
Hs578T
 CSCs
MCF7
CSCs
Sample#5
 CSCs
shPLA2G16-1
shPLA2G16-2
shPLA2G16-1
shPLA2G16-2
shPLA2G16-1
shPLA2G16-2
shPLA2G16-1
shPLA2G16-2
shCtrl
shCtrl
shCtrl
shCtrl
kDa
-18
PLA2G16
b-Actin
-42
E
Non-BCSCs
LEV
LV-PLA2G16
12
**
**
9
**
**
 Relative expression
 of PLA2G16
6
3
0
Sample
 #5
MCF7
BT549
Hs578T
F
 BT549
 non-CSCs
 Hs578T
non-CSCs
 MCF7
non-CSCs
Sample#5
non-CSCs
LV-PLA2G16
LV-PLA2G16
LV-PLA2G16
LV-PLA2G16
LEV
LEV
LEV
LEV
kDa
-18
PLA2G16
-42
b-Actin
G
Cytoplasm
Nuclear
Hs578T
Hs578T
MCF7
MCF7
kDa
-18
PLA2G16
-113
PARP
-36
GAPDH

## Slide 8
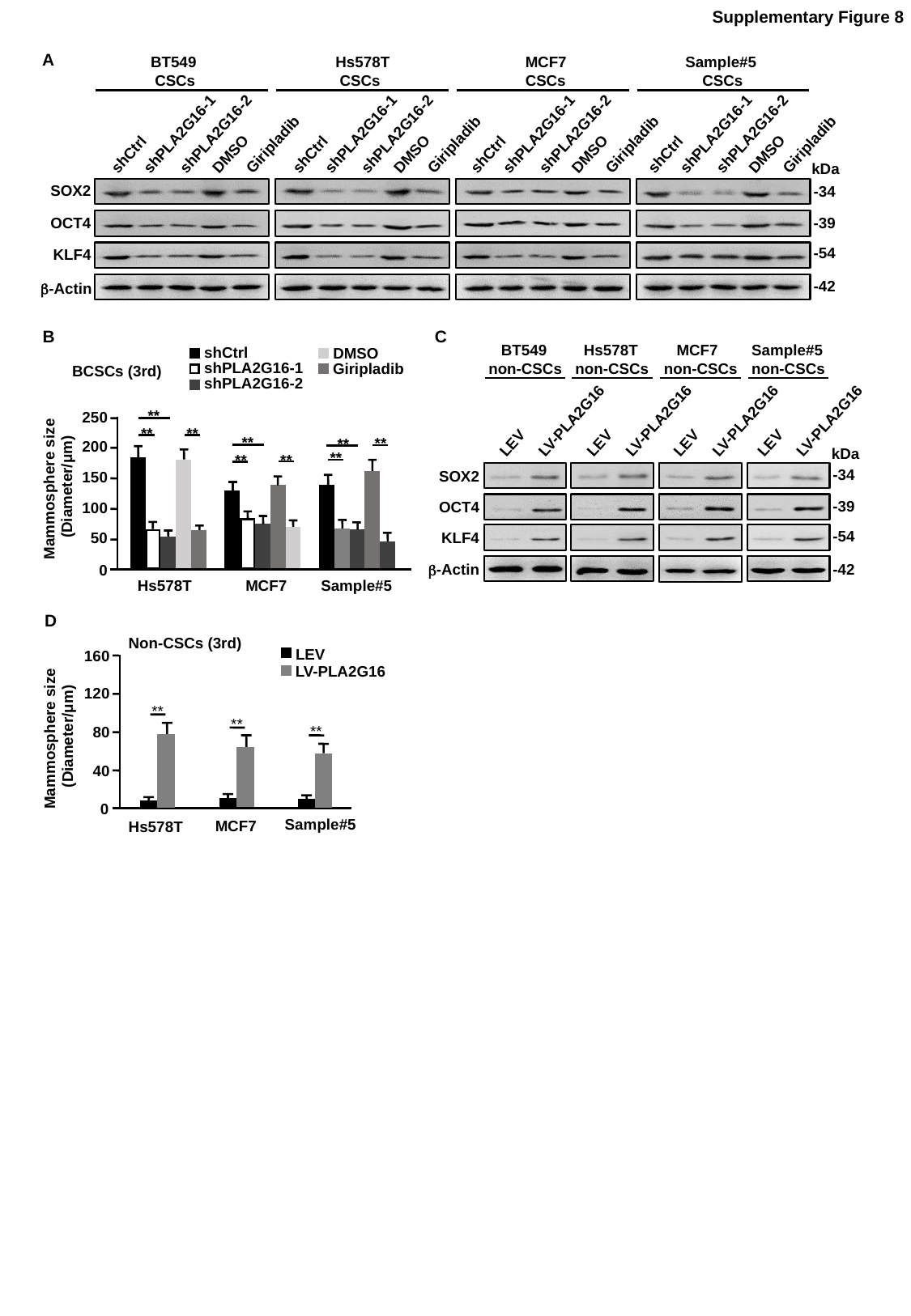

Supplementary Figure 8
A
BT549
 CSCs
Hs578T
 CSCs
MCF7
CSCs
Sample#5
 CSCs
shPLA2G16-1
shPLA2G16-2
shPLA2G16-1
shPLA2G16-2
shPLA2G16-1
shPLA2G16-2
shPLA2G16-1
shPLA2G16-2
Giripladib
Giripladib
Giripladib
Giripladib
shCtrl
DMSO
shCtrl
DMSO
shCtrl
DMSO
shCtrl
DMSO
kDa
SOX2
-34
OCT4
-39
-54
KLF4
-42
b-Actin
B
shCtrl
DMSO
shPLA2G16-1
Giripladib
shPLA2G16-2
BCSCs (3rd)
 **
250
 **
 **
 **
 **
 **
200
 **
 **
 **
Mammosphere size
 (Diameter/μm)
150
100
50
0
Hs578T
MCF7
Sample#5
C
 BT549
 non-CSCs
 Hs578T
non-CSCs
 MCF7
non-CSCs
Sample#5
non-CSCs
LV-PLA2G16
LV-PLA2G16
LV-PLA2G16
LV-PLA2G16
LEV
LEV
LEV
LEV
kDa
-34
SOX2
-39
OCT4
-54
KLF4
-42
b-Actin
D
Non-CSCs (3rd)
LEV
LV-PLA2G16
160
120
**
Mammosphere size
 (Diameter/μm)
**
**
80
40
0
 Sample#5
 MCF7
 Hs578T

## Slide 9
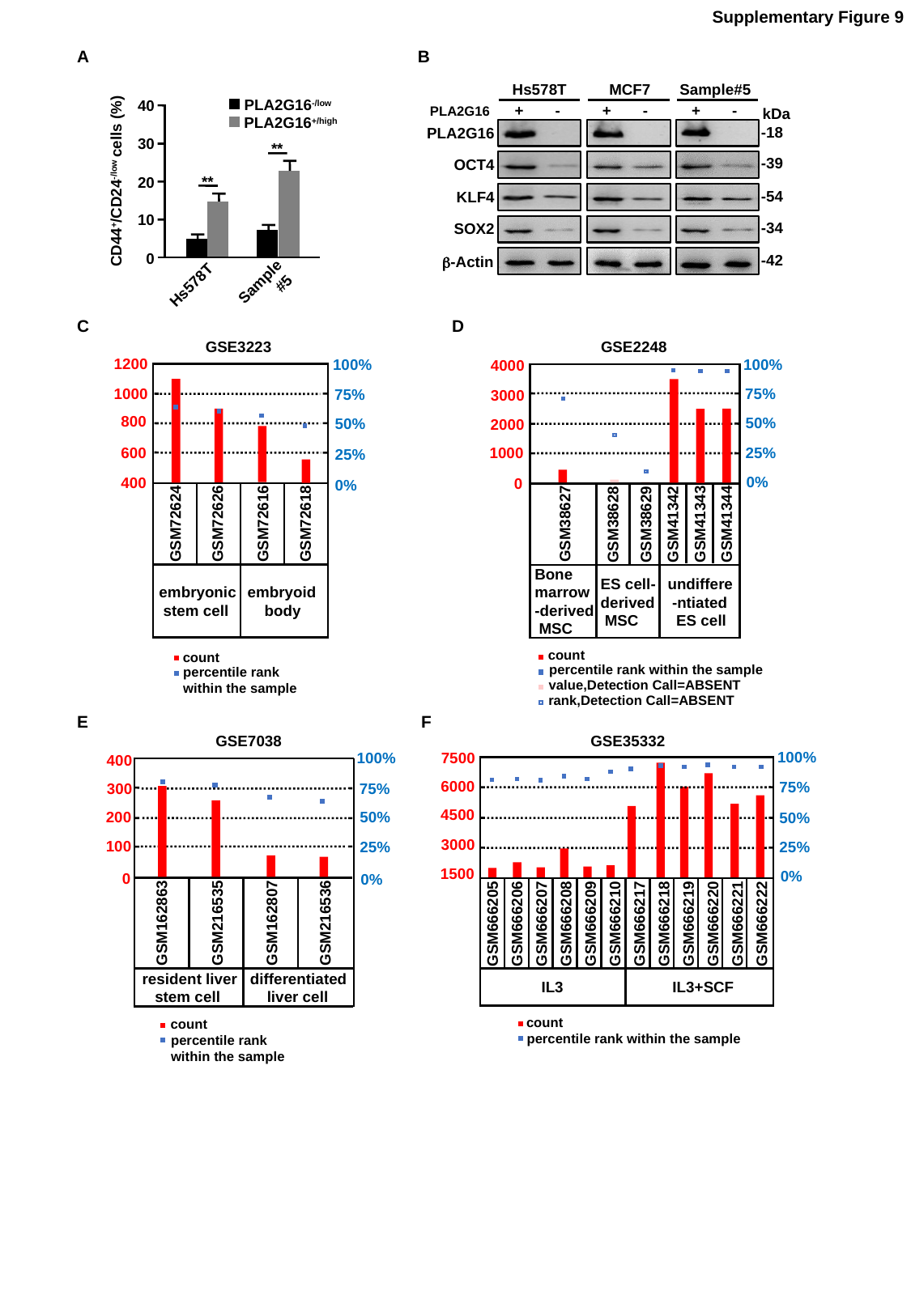

Supplementary Figure 9
A
PLA2G16-/low
PLA2G16+/high
40
30
**
**
20
CD44+/CD24-/low cells (%)
10
0
Sample
 #5
Hs578T
B
Hs578T
MCF7
Sample#5
+/
-
+
-
+
-
PLA2G16
kDa
-18
PLA2G16
-39
OCT4
-54
KLF4
-34
SOX2
-42
b-Actin
C
D
GSE3223
1200
100%
1000
75%
800
50%
600
25%
400
0%
GSM72624
GSM72626
GSM72616
GSM72618
embryonic
 stem cell
embryoid
 body
count
percentile rank
within the sample
GSE2248
100%
4000
75%
3000
50%
2000
25%
1000
0%
GSM38627
GSM38628
GSM38629
GSM41342
GSM41343
GSM41344
0
Bone
marrow
-derived
 MSC
ES cell-
derived
 MSC
undiffere
 -ntiated
 ES cell
count
percentile rank within the sample
value,Detection Call=ABSENT
rank,Detection Call=ABSENT
E
F
GSE7038
100%
400
75%
300
200
50%
100
25%
0
0%
GSM162863
GSM216535
GSM162807
GSM216536
 resident liver
 stem cell
differentiated
 liver cell
count
percentile rank
within the sample
GSE35332
100%
7500
6000
75%
4500
50%
3000
25%
1500
0%
GSM666205
GSM666206
GSM666207
GSM666208
GSM666209
GSM666210
GSM666217
GSM666218
GSM666219
GSM666220
GSM666221
GSM666222
IL3+SCF
IL3
count
percentile rank within the sample

## Slide 10
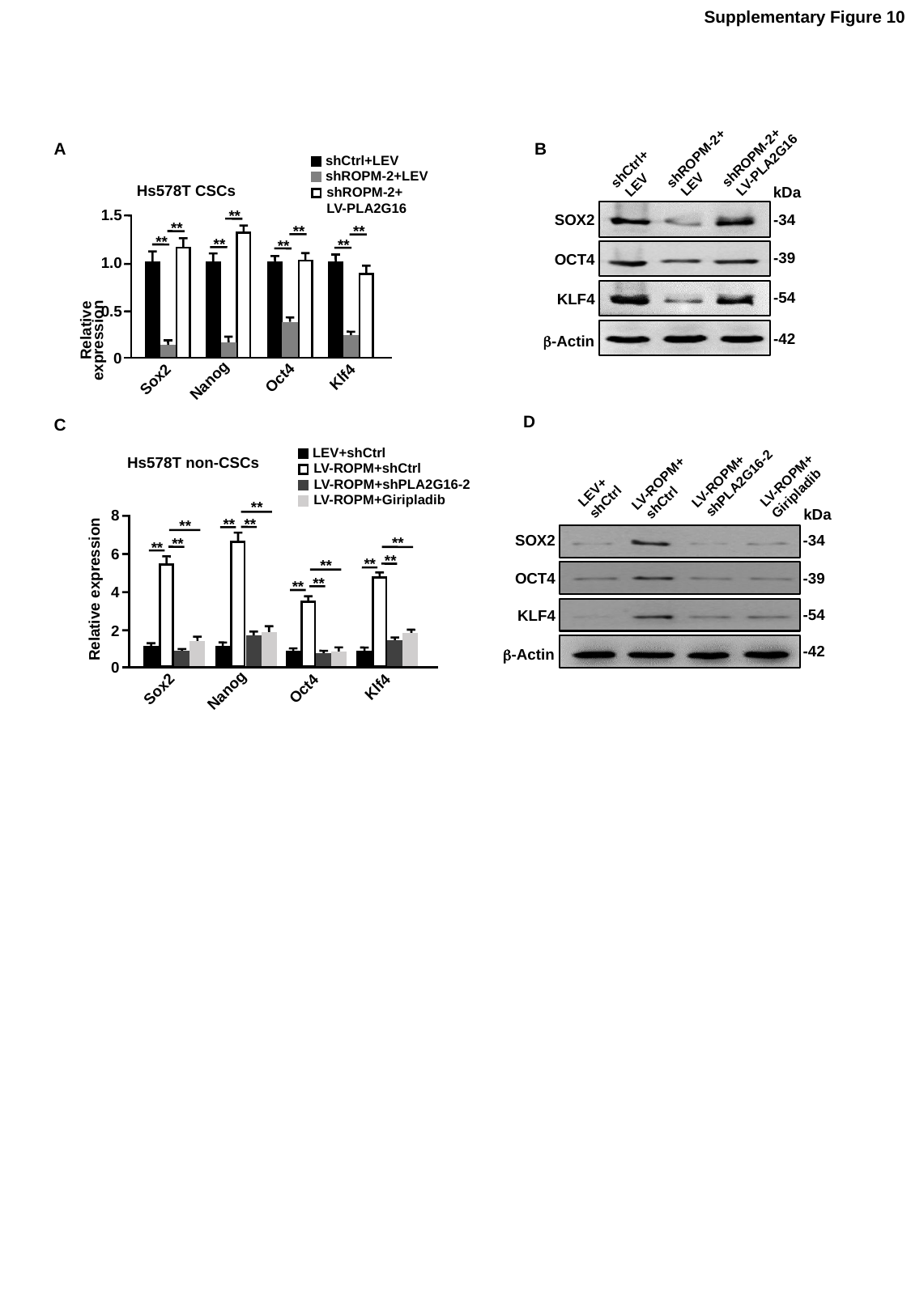

Supplementary Figure 10
A
B
shROPM-2+
 LV-PLA2G16
shROPM-2+
 LEV
shCtrl+
 LEV
kDa
-34
-39
-54
-42
SOX2
OCT4
KLF4
b-Actin
shCtrl+LEV
shROPM-2+LEV
shROPM-2+
LV-PLA2G16
Hs578T CSCs
**
**
1.5
**
**
**
**
**
**
1.0
 Relative expression
0.5
0
Klf4
Oct4
Sox2
Nanog
D
C
LEV+shCtrl
LV-ROPM+shCtrl
LV-ROPM+shPLA2G16-2
LV-ROPM+Giripladib
Hs578T non-CSCs
**
8
**
**
**
**
**
**
6
**
**
**
**
 Relative expression
**
4
2
0
Klf4
Sox2
Oct4
Nanog
 LV-ROPM+
 shPLA2G16-2
LV-ROPM+
Giripladib
LV-ROPM+
 shCtrl
LEV+
shCtrl
kDa
SOX2
-34
OCT4
-39
-54
KLF4
-42
b-Actin

## Slide 11
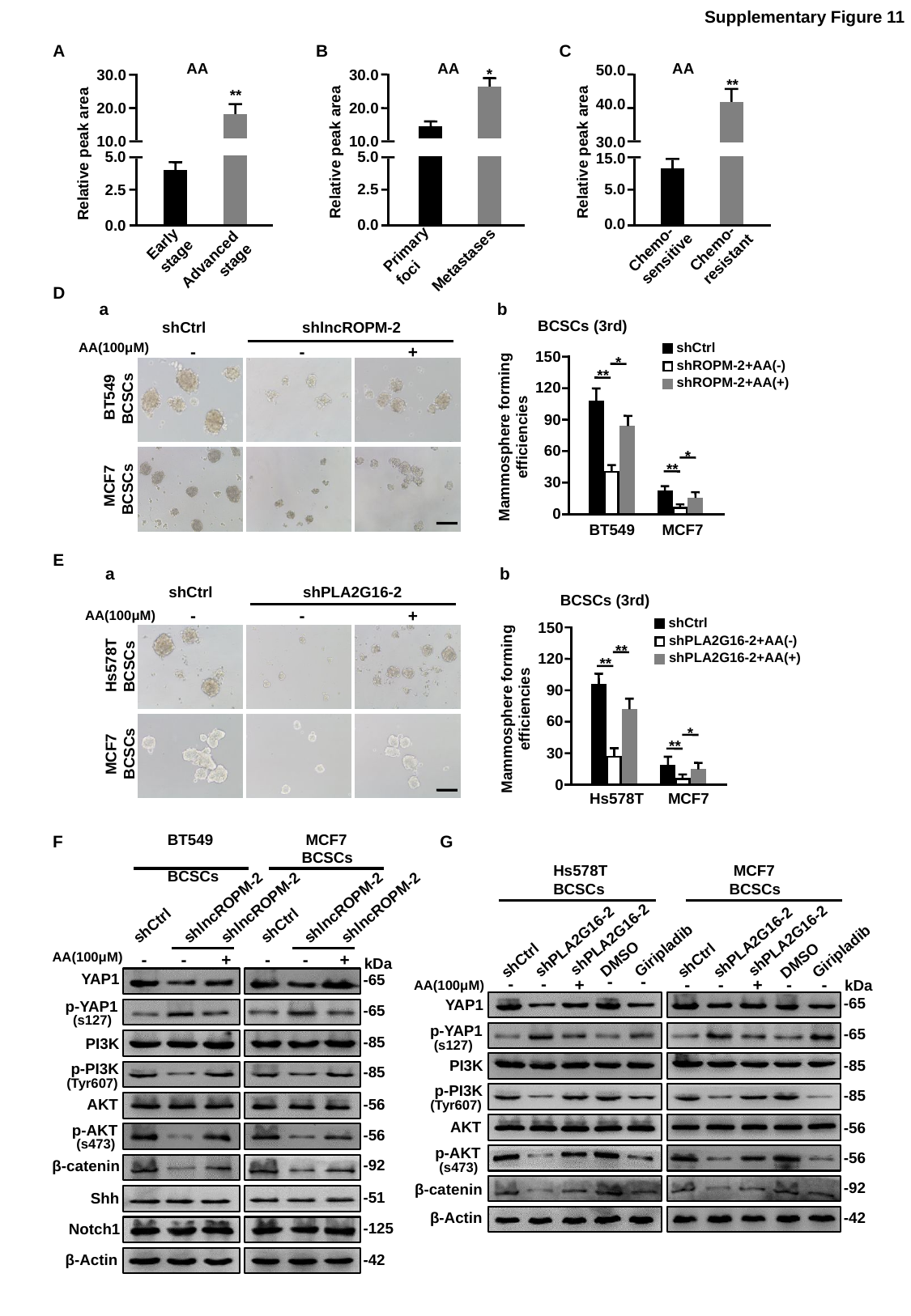

Supplementary Figure 11
A
B
C
AA
30.0
 **
20.0
10.0
5.0
Relative peak area
2.5
0.0
Early
stage
Advanced
 stage
AA
 *
30.0
20.0
10.0
Relative peak area
5.0
2.5
0.0
Primary
foci
Metastases
AA
50.0
 **
40.0
30.0
Relative peak area
15.0
5.0
0.0
Chemo-
sensitive
Chemo-
resistant
D
a
shCtrl
shlncROPM-2
AA(100μM)
-
-
+
 BT549 BCSCs
 MCF7 BCSCs
E
a
shCtrl
shPLA2G16-2
-
-
+
AA(100μM)
Hs578T BCSCs
 MCF7 BCSCs
b
BCSCs (3rd)
shCtrl
shROPM-2+AA(-)
shROPM-2+AA(+)
150
*
**
120
90
Mammosphere forming
 efficiencies
60
*
**
30
0
BT549
MCF7
b
BCSCs (3rd)
shCtrl
shPLA2G16-2+AA(-)
shPLA2G16-2+AA(+)
150
**
**
120
90
Mammosphere forming
 efficiencies
60
*
**
30
0
Hs578T
MCF7
BT549 BCSCs
 MCF7 BCSCs
shlncROPM-2
shlncROPM-2
shlncROPM-2
shlncROPM-2
shCtrl
shCtrl
AA(100μM)
-
-
+
-
-
+
kDa
YAP1
-65
-65
p-YAP1
 (s127)
-85
PI3K
-85
 p-PI3K
(Tyr607)
AKT
-56
p-AKT
 (s473)
-56
-92
β-catenin
-51
Shh
-125
Notch1
β-Actin
-42
F
G
Hs578T BCSCs
 MCF7 BCSCs
shPLA2G16-2
shPLA2G16-2
shPLA2G16-2
shPLA2G16-2
Giripladib
Giripladib
DMSO
DMSO
shCtrl
shCtrl
-
-
-
-
+
-
-
+
-
-
kDa
AA(100μM)
-65
YAP1
-65
p-YAP1
 (s127)
-85
PI3K
 p-PI3K
(Tyr607)
-85
AKT
-56
-56
p-AKT
 (s473)
-92
β-catenin
-42
β-Actin

## Slide 12
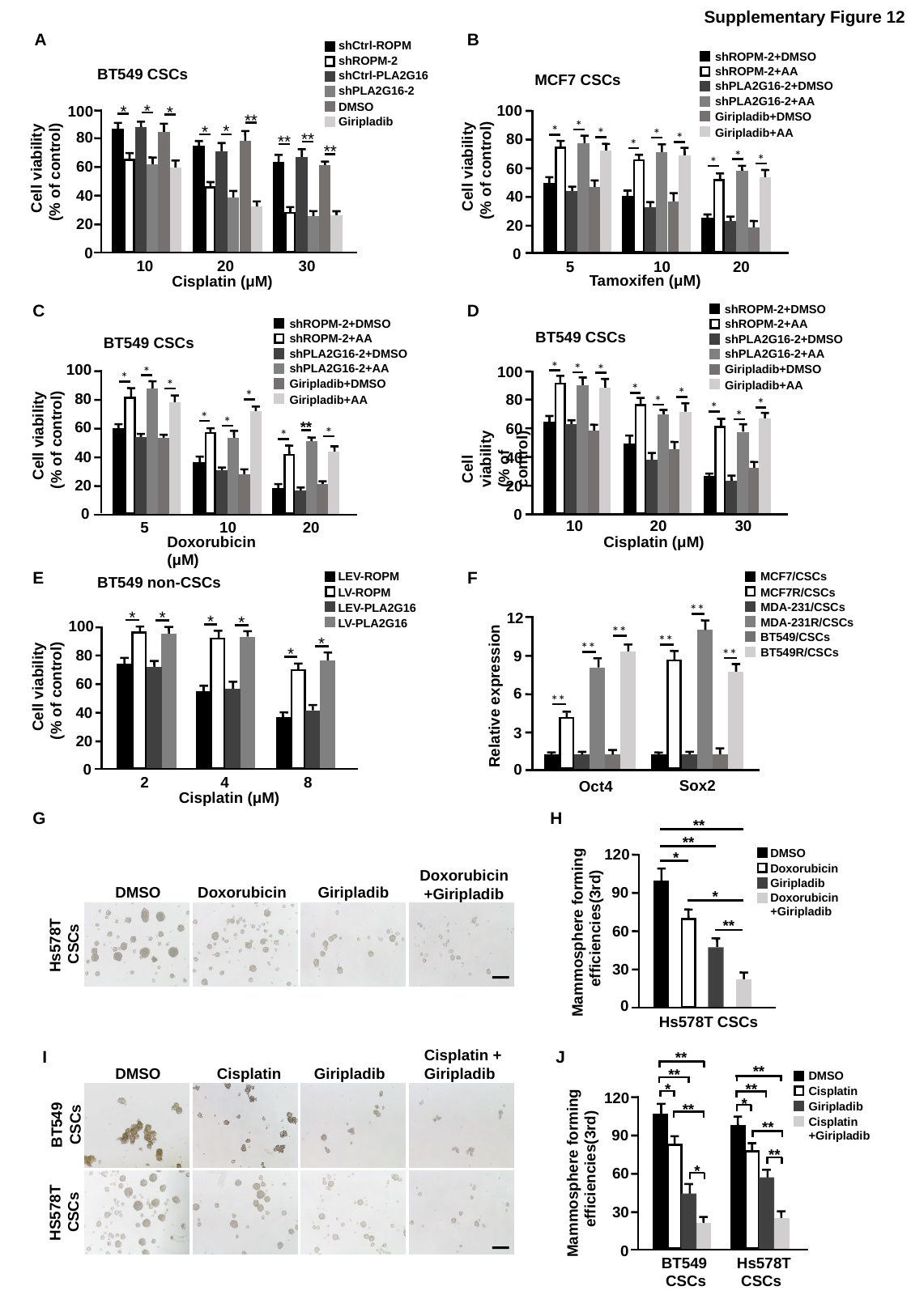

Supplementary Figure 12
A
B
shROPM-2+DMSO
shROPM-2+AA
shPLA2G16-2+DMSO
shPLA2G16-2+AA
Giripladib+DMSO
Giripladib+AA
MCF7 CSCs
 Cell viability
 (% of control)
100
*
*
*
*
*
80
*
*
*
*
60
40
20
0
5
10
20
Tamoxifen (μM)
shCtrl-ROPM
shROPM-2
shCtrl-PLA2G16
shPLA2G16-2
DMSO
Giripladib
BT549 CSCs
*
*
*
 Cell viability
 (% of control)
100
**
*
*
**
**
80
**
60
40
20
0
10
20
30
Cisplatin (μM)
C
shROPM-2+DMSO
shROPM-2+AA
shPLA2G16-2+DMSO
shPLA2G16-2+AA
Giripladib+DMSO
Giripladib+AA
BT549 CSCs
*
100
*
 Cell viability
 (% of control)
*
*
80
*
*
 **
*
60
*
40
20
0
5
10
20
Doxorubicin (μM)
D
shROPM-2+DMSO
shROPM-2+AA
shPLA2G16-2+DMSO
shPLA2G16-2+AA
Giripladib+DMSO
Giripladib+AA
BT549 CSCs
*
*
*
100
*
*
*
*
 Cell viability
(% of control)
80
*
*
60
40
20
0
10
20
30
Cisplatin (μM)
E
F
MCF7/CSCs
MCF7R/CSCs
MDA-231/CSCs
MDA-231R/CSCs
BT549/CSCs
BT549R/CSCs
**
12
**
**
**
**
9
Relative expression
6
**
3
0
Sox2
Oct4
LEV-ROPM
BT549 non-CSCs
LV-ROPM
 Cell viability
 (% of control)
LEV-PLA2G16
*
*
*
*
LV-PLA2G16
100
*
*
80
60
40
20
0
2
4
8
Cisplatin (μM)
G
H
**
**
DMSO
Doxorubicin
Giripladib
Doxorubicin +Giripladib
*
120
*
90
**
Mammosphere forming
 efficiencies(3rd)
60
30
0
Hs578T CSCs
J
**
**
**
DMSO
Cisplatin
Giripladib
Cisplatin
+Giripladib
**
*
*
120
 **
**
90
**
Mammosphere forming
 efficiencies(3rd)
*
60
30
0
BT549
 CSCs
Hs578T
 CSCs
Doxorubicin
 +Giripladib
DMSO
Doxorubicin
Giripladib
Hs578T
 CSCs
Cisplatin +
Giripladib
DMSO
Cisplatin
Giripladib
 BT549
 CSCs
HS578T
 CSCs
I
