# Supplementary material for: A novel lncRNA ROPM-mediated lipid metabolism governs breast cancer stem cell properties
Source: J Hematol Oncol. 2021 Oct 29;14:178. doi: 10.1186/s13045-021-01194-z (PMC8555326; doi:10.1186/s13045-021-01194-z)
Supplement: Supplementary file 2 — Additional file 2. Oligomers used in this study. [file 13045_2021_1194_MOESM2_ESM.pptx]

## Slide 1
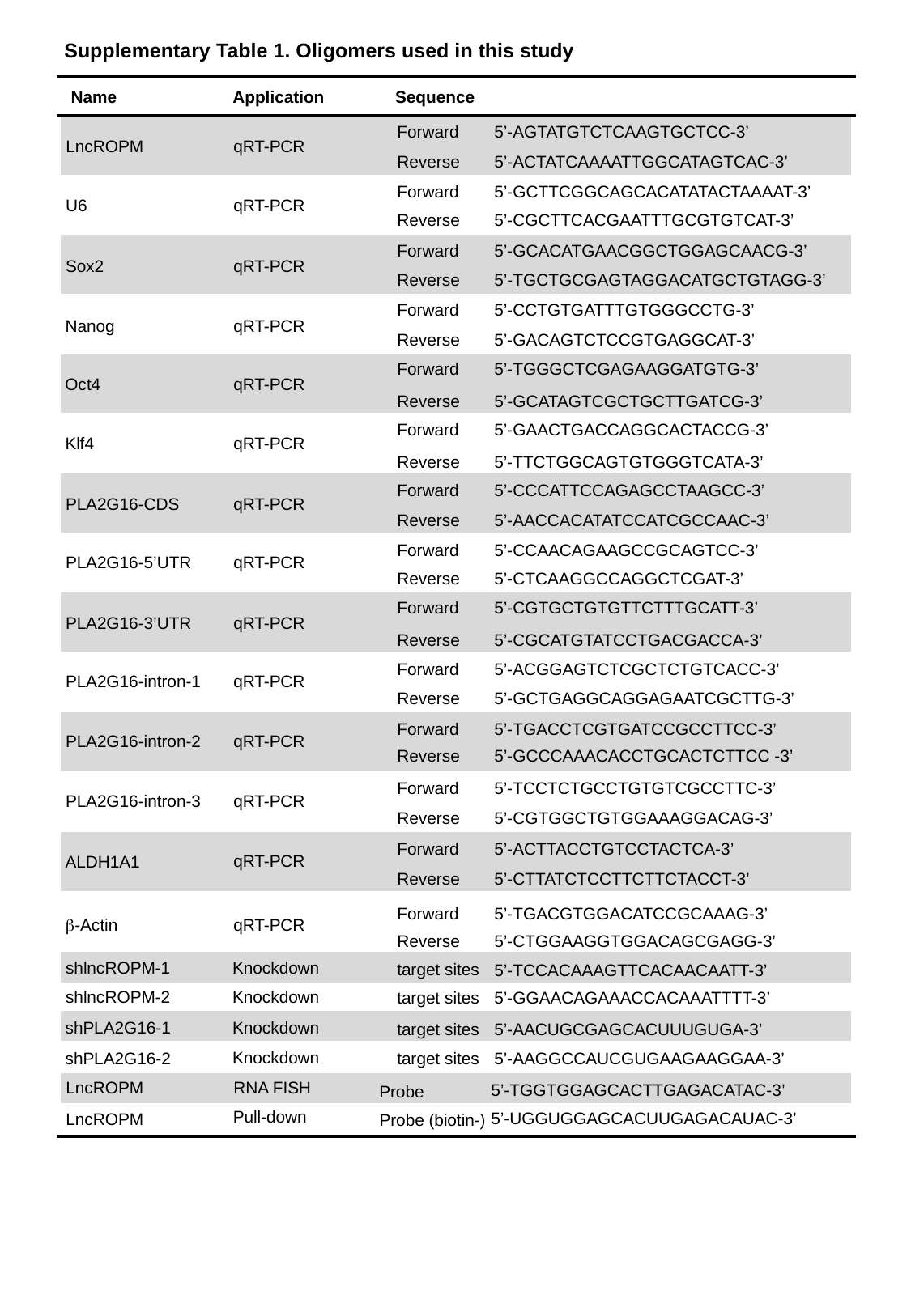

Supplementary Table 1. Oligomers used in this study
Name
Application
Sequence
 Forward
5’-AGTATGTCTCAAGTGCTCC-3’
LncROPM
qRT-PCR
 Reverse
5’-ACTATCAAAATTGGCATAGTCAC-3’
 Forward
5’-GCTTCGGCAGCACATATACTAAAAT-3’
U6
qRT-PCR
 Reverse
5’-CGCTTCACGAATTTGCGTGTCAT-3’
 Forward
5’-GCACATGAACGGCTGGAGCAACG-3’
Sox2
qRT-PCR
 Reverse
5’-TGCTGCGAGTAGGACATGCTGTAGG-3’
 Forward
5’-CCTGTGATTTGTGGGCCTG-3’
Nanog
qRT-PCR
 Reverse
5’-GACAGTCTCCGTGAGGCAT-3’
 Forward
5’-TGGGCTCGAGAAGGATGTG-3’
Oct4
qRT-PCR
 Reverse
5’-GCATAGTCGCTGCTTGATCG-3’
 Forward
5’-GAACTGACCAGGCACTACCG-3’
Klf4
qRT-PCR
 Reverse
5’-TTCTGGCAGTGTGGGTCATA-3’
 Forward
5’-CCCATTCCAGAGCCTAAGCC-3’
PLA2G16-CDS
qRT-PCR
 Reverse
5’-AACCACATATCCATCGCCAAC-3’
 Forward
5’-CCAACAGAAGCCGCAGTCC-3’
PLA2G16-5’UTR
qRT-PCR
 Reverse
5’-CTCAAGGCCAGGCTCGAT-3’
 Forward
5’-CGTGCTGTGTTCTTTGCATT-3’
PLA2G16-3’UTR
qRT-PCR
 Reverse
5’-CGCATGTATCCTGACGACCA-3’
 Forward
5’-ACGGAGTCTCGCTCTGTCACC-3’
PLA2G16-intron-1
qRT-PCR
 Reverse
5’-GCTGAGGCAGGAGAATCGCTTG-3’
 Forward
5’-TGACCTCGTGATCCGCCTTCC-3’
PLA2G16-intron-2
qRT-PCR
 Reverse
5’-GCCCAAACACCTGCACTCTTCC -3’
 Forward
5’-TCCTCTGCCTGTGTCGCCTTC-3’
PLA2G16-intron-3
qRT-PCR
 Reverse
5’-CGTGGCTGTGGAAAGGACAG-3’
 Forward
5’-ACTTACCTGTCCTACTCA-3’
qRT-PCR
ALDH1A1
 Reverse
5’-CTTATCTCCTTCTTCTACCT-3’
 Forward
5’-TGACGTGGACATCCGCAAAG-3’
b-Actin
qRT-PCR
 Reverse
5’-CTGGAAGGTGGACAGCGAGG-3’
 target sites
5’-TCCACAAAGTTCACAACAATT-3’
shlncROPM-1
Knockdown
 target sites
5’-GGAACAGAAACCACAAATTTT-3’
shlncROPM-2
Knockdown
 target sites
5’-AACUGCGAGCACUUUGUGA-3’
shPLA2G16-1
Knockdown
 target sites
5’-AAGGCCAUCGUGAAGAAGGAA-3’
Knockdown
shPLA2G16-2
5’-TGGTGGAGCACTTGAGACATAC-3’
 Probe
LncROPM
RNA FISH
5’-UGGUGGAGCACUUGAGACAUAC-3’
 Probe (biotin-)
Pull-down
LncROPM
